# Evidence of mitochondria origin of SARS-CoV-2 double-membrane vesicles: a review.

**DOI:** 10.12688/f1000research.73170.1

**Published:** 2021-10-05

**Authors:** Pavel Montes de Oca-B

**Affiliations:** 1 Neurociencia Cognitiva, Instituto de Fisiologia-UNAM, CDMX, CDMX, 04510, Mexico; 2 Unidad de Neurobiologia Dinamica, Instituto Nacional de Neurologia y Neurocirugia, CDMX, CDMX, 14269, Mexico

**Keywords:** SARS CoV-2; DMV; MDV; mitochondria; caveolae; COVID

## Abstract

The coronavirus disease-19 (COVID-19) pandemic is caused by the coronavirus, SARS-CoV-2, which has infected in a year more than 200 million people and has killed almost 4.5 million people worldwide. This infection affects mainly certain groups of people that have high susceptibility to present severe COVID-19 due to comorbidities. Moreover, long-COVID-19 comprises a series of symptoms that may remain in some patients for months after infection that further compromises health of individuals. Therefore, this pandemic poses a serious emergency worldwide. Thus, since this pandemic is profoundly affecting economic and social life of societies, a deeper understanding of SARS-CoV-2 infection cycle could help to envisage novel therapeutic alternatives that limit or stop COVID-19.

Several recent findings have unexpectedly found that mitochondria play a critical role in SARS-CoV-2 cell infection. Indeed, it has been suggested that this organelle could be the origin of its replication niches, the double membrane vesicles (DMV), as it has been observed with other virus. In this regard, mitochondria derived vesicles (MDV), involved in mitochondria quality control, were discovered more than 10 years ago and, interestingly, there is a population characterized by a double membrane. MDV shedding is induced by mitochondrial stress and it has a fast assembly dynamic, reason that perhaps has precluded their identification in electron microscopy or tomography studies. These and other features of MDV together with recent SARS-CoV-2 protein interactome with the host and other findings linking SARS-CoV-2 to mitochondria, support that these vesicles are the precursors of SARS-CoV-2 induced DMV. In this work, the celular, molecular phenotypical and biochemical evidence that supports this hypothesis is reviewed and integrated into the current model of SARS-CoV-2 cell infection. In this scheme, some relevant questions are raised as pending topics for research that would help in the near future to test this hypothesis. The intention of this work is to provide a novel framework that could open new possibilities to tackle SARS-CoV-2 pandemic through mitochondria targeted therapies.

## Background

The coronavirus disease-19 (COVID-19) pandemic is caused by the positive-sense single-stranded RNA coronavirus, SARS-CoV-2, that has infected, in a year, more than 200 million people and has killed almost 4.5 million people worldwide ^1^ since it has no definitive and effective treatment until today. This infection affects mainly certain groups of people that have high susceptibility to present severe COVID-19 due to comorbidities that include cardiovascular disease, chronic kidney, respiratory or liver disease, severe obesity, or hypertension among others. In these patients, the cytokine storm induced by the virus poses a serious death risk for these patients due to the systemic inflammation and multiorgan failure ^2– 6^. Moreover, the so-called long COVID-19 comprises a series of symptoms that may remain in some patients for months after infection that further compromises their health, even after non-severe COVID-19 ^7^. Despite huge efforts to stop infections and deaths worldwide, only a few treatments have been demonstrated to ameliorate severe COVID-19, and different vaccine strategies are currently under investigation in clinical phase III and/or IV trials. Therefore, in this scenario, a deeper understanding of the cellular mechanisms exploited by SARS-CoV-2 for cell infection could undoubtedly provide new unforeseen strategies to tackle this pandemic.

## The SARS-CoV-2 replication organelles and the unresolved origin of double-membrane vesicles

Several recent reports have shown that mitochondria play a relevant role during SARS-CoV-2 infection ^8– 10^. These findings and previously published results of SARS-CoV-2, SARS-CoV and other coronavirus biology allow us to hypothesize that mitochondria could be responsible for the assembly of double-membrane vesicles (DMV). These are membrane modifications induced by SARS-CoV-2 and it is where viralRNA (vRNA) replication occurs in the infected cell, that are believed to be derived from the endoplasmic reticulum (ER) or other mechanisms, such as autophagy ^11– 13^. However, some published literature supports that double-membrane mitochondria-derived vesicles (MDV), discovered some years ago ^14^, could be the precursors or relatives of DMV. This hypothesis of mitochondria role in DMV assembly and the involvement of MDV has been suggested previously ^8, 15, 16^. Indeed, specialized replication organelles (RO) at mitochondria outer membrane (MOM) have been observed in FHV ^17^, whereas HIV RNA is known to locate in the mitochondria ^18^. Here, a brief review of the evidence that supports this notion is presented and integrated into the current model ^19^, with the intention to provide a novel framework that could open possibilities to tackle the SARS-CoV-2 pandemic.

**Figure 1.**
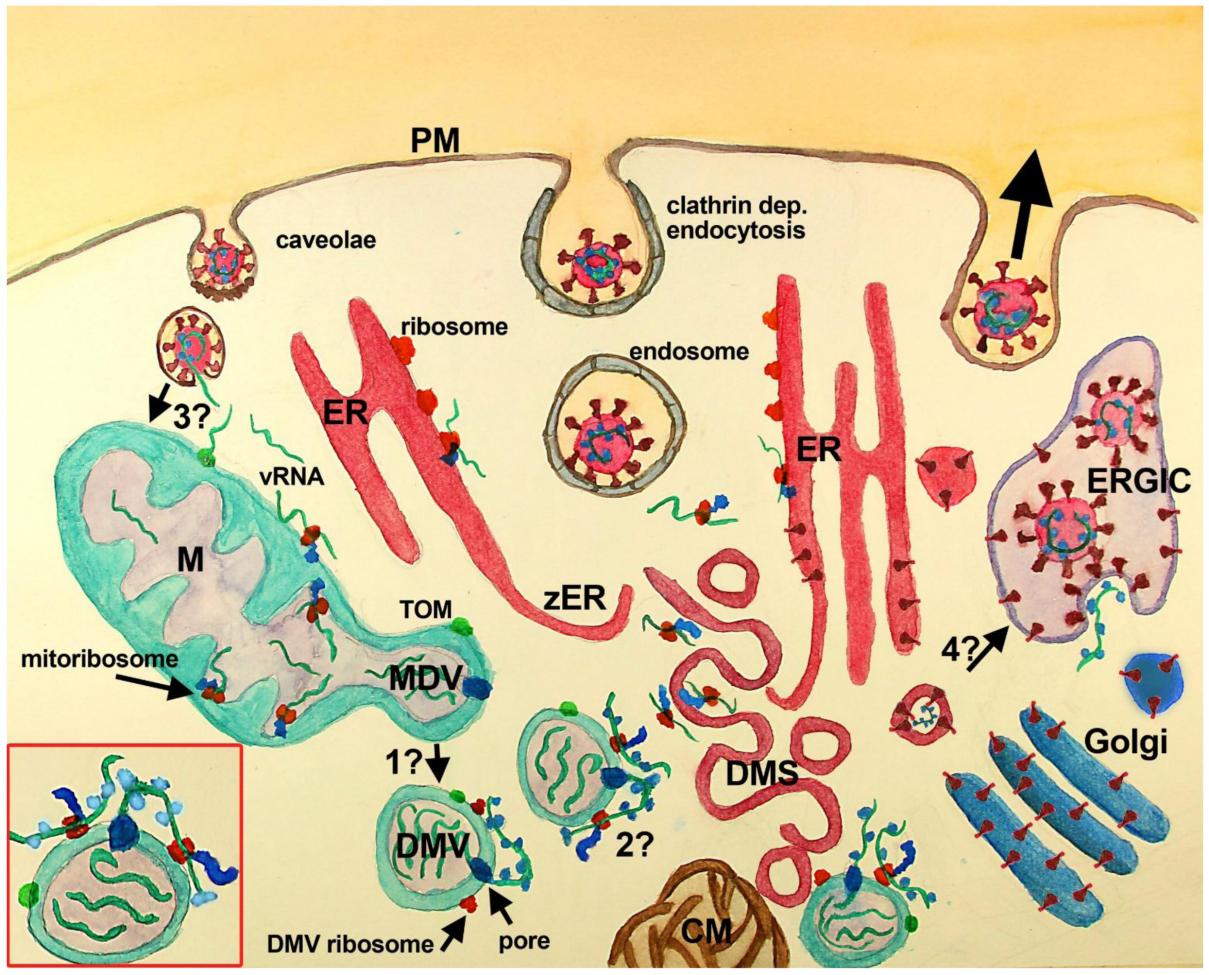
Scheme of SARS-CoV-2 replication cycle with mitochondria and MDV participation. SARS Cov2 recognizes ACE2 (not shown) at the PM of the host cell inducing its endocytosis. Several other entry factors and facilitators have been found to mediate SARS-CoV-2 entry into the host cells. Clathrin-dependent and independent endocytosis mediate viral entry. **In the current paradigm**, clathrin-mediated endocytosis (middle vesicle at PM) follows the endosomal pathway, that through endosomal acidification and cleavage of S protein by TMPRS2 induces the fusion of SARS-CoV-2 membrane with vesicle membrane deploying vRNA (green stripes) into the cytoplasm. Once released, vRNA reaches cytoplasmic and ER ribosomes starting viral protein synthesis. Viral proteins induce the assembly of RO elements that are interconnected (not shown), initially inducing zER that through bending and scission assemble DMS, CM, and probably DMV, where synthesis of viral proteins and vRNA takes place. Proteins synthesized at ER, DMS and CM reach the Golgi, where they are posttranslationally modified, and the ERGIC, where the viral particles are assembled and set ready for exocytosis (large black arrow, right vesicle at PM). Viral particles have also been observed at multivesicular bodies (not shown). In this paradigm, DMV are believed to derive from DMS and/or CM, although some controversies have been raised (see text), mainly the temporal sequence, the lack of ER markers in DMV and the lack of intermediate structures. VP (not shown) are formed by the fusion of single DMS. In this paradigm, the mitochondrial role is not considered, although evidence has accumulated suggesting its participation (see text). **In the complementary scenario** proposed in this work, DMV are shedded from mitochondria, through a mechanism similar to that described for double-membrane MDV, and or asymmetric mitochondria fission, known to be potentiated after mitochondrial stress. Whether MDV require several transforming steps to become DMV, or if these compartments are essentially the same with viral proteins included needs further investigation ( **question mark 1**). DMV have double-membrane spanning pores (dark blue) in which nsp3 is inserted along with other unidentified molecules. These pores mediate the export of vRNA to the cytoplasm, which complexes with N protein outside DMV. Exported vRNA may be translated immediately by ribosomes located in the external membrane of DMV ( **question mark 2**). Interestingly, some MDV have been shown to carry mitochondrial proteins of the IMM, MOM, and mitochondria matrix, which could be also present at DMV (green dot at MOM, MDV/DMV outer membrane). In this complimentary scenario, a critical question is how vRNA accesses mitochondria ( **question mark 3**). It is possible that vRNA once in the cytoplasm is translated at MOM ribosomes, or that it is translocated into mitochondria through the TOM complex. Alternatively, vRNA could reach mitochondria through the fusion of caveolae with endocytosed SARS-CoV-2, although it is not clear whether coronavirus can follow this pathway. Finally, an intriguing possibility, that could be critical, is whether DMS, which are induced by viral proteins most probably synthesized nearby the ER from where they are derived, may transform into vesicles with viral particles. This possibility is supported by the synthesis of viral proteins at DMS and would require that vRNA is packed inside, which seems feasible because protein synthesis at DMS implicates the presence of vRNA. The lack of *de novo* synthesized vRNA at DMS indicates that vRNA synthesis does not happen there, but does not rule out the presence of vRNA within DMS. **Inset, red square left bottom:** A close-up of a DMV with vRNA in its interior (3 green stripes), with one vRNA being exported by the double-membrane spanning pore (dark blue at DMV membranes), complexing with the N protein (light blue). This exported vRNA could be translated in situ at DMV ribosomes (red at DMV external membrane). Peptides synthesized by DMV ribosomes are shown attached in blue and the green dot at DMV represents mitochondrial molecules located at DMV. **Abbreviations**: **CM**-convoluted membranes; **DMS**-double-membrane spherules; **DMV**-double-membrane vesicles; **ER**-endoplasmic reticulum; **ERGIC**-ER-Golgi intermediate compartment; **M**-mitochondria; **PM**-plasma membrane; **TOM**-translocase of the mitochondrial outer membrane; **VP**-vesicle packet (not shown); **vRNA**-viral RNA; **zER**-zippered ER.

DMV along with other membrane modifications are part of the RO induced by SARS-CoV-2 that also includes convoluted membranes (CM), zippered ER (zER), vesicle packets (VP), and double-membrane spherules (DMS) ^19^ ( Figure 1). RO are induced by SARS-CoV-2 in infected cells, as a variety of RO are induced by viruses including other nidovirus and picornavirus among others ^11, 13^. DMV assembly is induced by viral proteins but seems to also require other viral or host factors because cell plasmid transfection of transmembrane-containing non-structural proteins (nsp) 3-, 4-, and 6-induced membrane arrangements that resemble DMV but with smaller size ^20^. These nsp are part of the complex involved in vRNA replication, together with nsp12, the RNA-dependent RNA polymerase, and other nsp. Consistently, nsp4 mutation alters the assembly of DMVs ^15^ and abolishes viral replication ^21^. Nsp4, 3, and the nuclear (N) protein are located at the DMV ^16, 19^, where nsp3 has been shown to form a pore complex that communicates DMV interior with the cytoplasm, that was elusive for some time, pore that could also involve host factors and/or other viral proteins ^22^.

Most SARS-CoV-2-induced membrane modifications are derived from ER membranes and are interconnected. In the case of DMV, this origin was in part assumed because such mechanism is presumed to mediate RO assembly in other positive sense RNA viruses ^23^; because they have contacts with other membrane modifications of the RO and the ER, because ribosomes have been observed on DMV surface, and because DMS and CM were proposed to be precursors of DMV ^11, 16, 19, 20, 23^. DMV sizes range from 150 to 300 nm, but they grow through infection, and it has been established that SARS-CoV-2 DMV are the location where vRNA synthesis occurs ^16, 19, 24^. Importantly, DMV are early (1–2 h post-infection [p.i.]) observed in the cell cytoplasm after coronavirus infection *in vitro*, and their number increase through time reaching a maximum in 6–8 h p.i. ^16, 25^. There are currently two models for DMV assembly from the ER. In the case of coronavirus, DMV are thought to be assembled from zippered ER that folds and closes in response to vRNA, as observed with IBV ^11, 23^, with CM and DMS as putative intermediate precursors ^16, 19^. However, major challenges remain for this model, because no intermediate structures have been recognized between DMV and DMS or CM, and because CM and DMS, both derived from the ER, have no relationship with DMV beyond their membrane contacts ^16, 19^. DMV do not have ER (nor ERGIC, Golgi, or endosomal/lysosomal) markers as it would be expected if they were assembled from the ER, and most importantly, it has been shown that DMV assembly (1–2 h p.i.) precedes CM assembly (~3 h p.i.) ^25^. Furthermore, CM and DMS do not carry out vRNA synthesis as DMV do ^19^. Thus, CM and DMS are not DMV precursors ^19^. On the other hand, the high energetic and complex topological requirements assumed to occur for DMV assembly through the zippered ER−CM−DMS model, given their numbers after a few hours post-infection, further complicate this notion. Indeed, it has been suggested that DMV could have another origin than the ER ^8, 11, 19^.

## The mitochondria and double-membrane MDV as the putative origin of SARS-CoV-2 DMV

**Figure 2.**
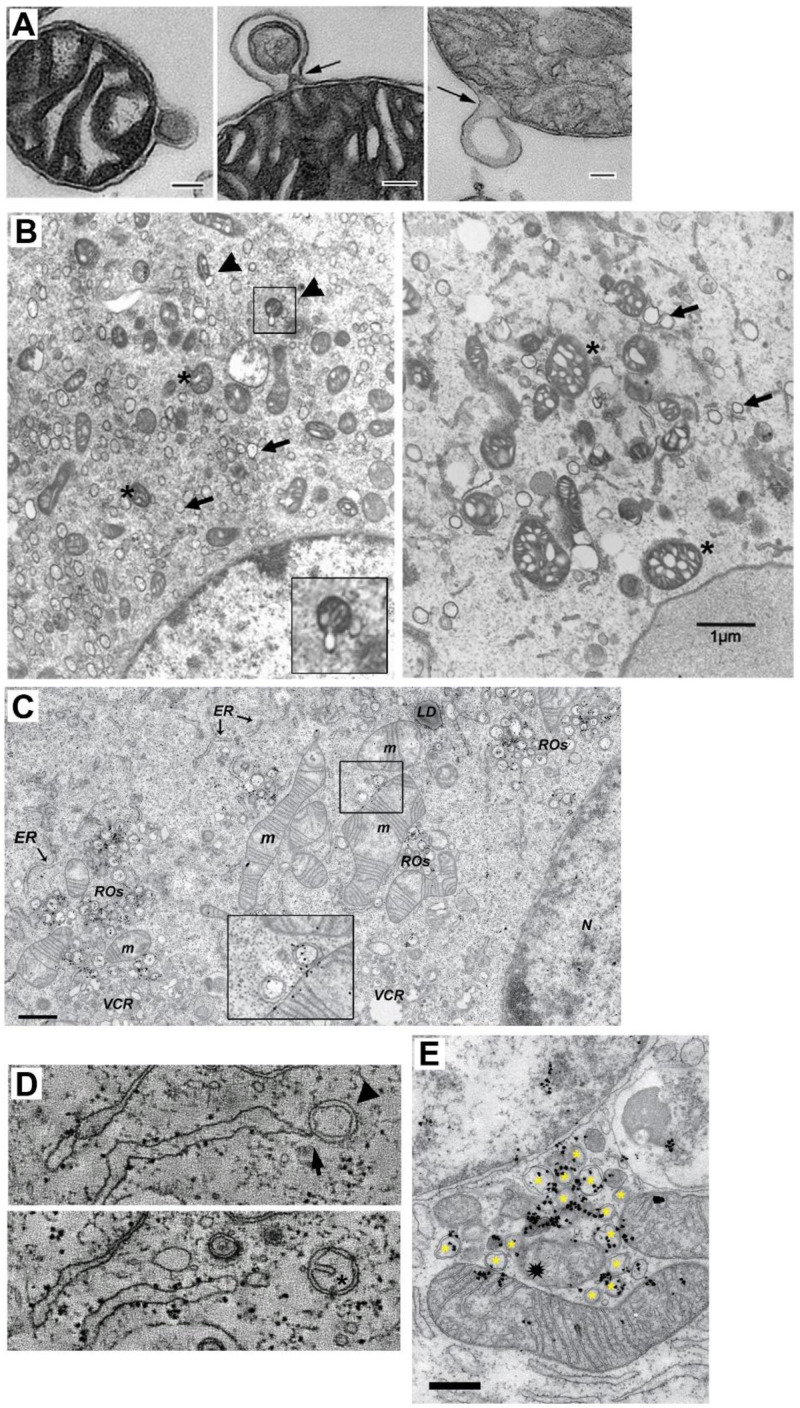
Double membrane MDV and DMV-mitochondria relationship. **A** TEM of three isolated mitochondria from bovine heart shedding double membrane vesicles (bar=500 nm in first panel and =100 nm in panels 2 and 3). (Taken ftom 29). **B** TEM analysis of mitochondria morphology and DMV formation in infectious clone virus (icv)-infected cells (derived from coronavirus murine hepatitis virus; MHV). This icv has a mutated nsp4-N258T that results in temperature sensitive viral replication. As observed, after 5.5 h p.i at the pemissive temperature, DMV are evident (arrows) and some mitochodria (asterisk) are associated with shedded vesicles (left panel, arrowheads and inset). Interestingly, if cells are left at the non-permissive temperature the last 2 h. (right panel), mitochondria appear swollen with enlarged cisternae, and accompanied by increased localization of nsp3 and nsp4 at mitochondria (not shown).(Modified from 15 with permission) (bar=1000 nm). **C** TEM and autoradiography of *de novo* synthesized vRNA in SARS CoV infected-Vero E6 cells at 7 h p.i. As observed, some DMV are closely located to mitochondria, showing both vRNA label within. Interestingly, vRNA label in DMV and mitochondria are neighboring (inset) (Modified from 19). **D** Electron tomography images of an Infectious Bronchitis Virus-infected cell showing a DMV (arrowhead) connected to the ER (arrow) (upper panel). In a different plane (lower panel), the same DMV shows a cisternae-like arrangement of the inner membrane (asterisk), that resembles those of mitochondria, hinting the putative mitochondrial origin of DMV (Modified from 23 with permission). **E** TEM and autoradiography of *de novo* synthesized vRNA in SARS-CoV-infected Vero E6 cells at 12 h p.i. It can be observed that DMV (asterisks) are densely labeled with vRNA signal. Notably, a couple of mitochondria are also labeled for vRNA, but importantly, this label is polarized in both mitochondria near the membrane towards the DMV cluster. Intriguilgly, within this pool of DMV, a degenerated mitochondria-like structure is observed (spark at the center), perhaps resulting from extensive DMV shedding (bar=500 nm) (Modified from 19).

Given the recent findings that relate SARS-CoV-2 with mitochondria, it is possible that MDVs are the origin of DMV. MDV were discovered more than a decade ago and they comprise different vesicles shed from mitochondria involved in its quality control ^14, 26^. Interestingly, some MDV have double-membrane with a size of 60–150 nm and are shed independently of drp1, mitochondria fission, and autophagy ( Figure 2A) ^26, 27^. Interestingly, several coronavirus proteins have been shown to down- or up-regulate drp1 (N/envelope [E], nsp3, nsp4a, nsp4b, and ofr9b) ^10^. MDV are generated in steady conditions and have been observed *in vivo* ^28^, but their number increases after mitochondrial stress or higher respiratory activity ^26, 27^. In this regard, it has been found that SARS-CoV-2-infected monocytes have compromised mitochondrial function and energy deficit ^30, 31^. This could be the long-term result of viral infection in which mitochondria shedding of MDV/DMV, triggered initially by a burst of metabolic activity or stress, leads to mitochondria function impairment.

MDV not only look like DMV for their double-membrane, they also transport selective cargo to peroxisomes and the endolysosomal system after mitochondrial stress, depending on Vps35 and syntaxin-17/SNAP29/VAMP7, respectively ^26, 27, 32, 33^. This offers a pathway that could be involved in the intracellular transport of viral components to secondary vesicular structures ( *i.e.* ERGIC, lysosomes, multivesicular bodies) where viral particles are assembled and set ready for exocytosis. Interestingly, PINK1/Parkin and the mitochondrial ubiquitin ligase MULAN1 (MAPL) have been involved in MDV shedding from mitochondria ^27, 34^. Notably, MULAN1 is known to be involved in the antiviral response of mitochondria ^35^. In addition, some MDV contain mitochondrial molecules, including the translocase complex (specifically TOM 20), components of the OXPHOS complexes, the VDAC, and/or pyruvate dehydrogenase ^26, 29^. This opens the possibility that other mitochondrial components and resources for vRNA replication are transported or generated within MDV, such as proteins involved in the translocation of metabolites and solutes since MDV are like “chunks” of mitochondria. In this regard, it was recently shown that DMV have pores that span the double-membrane that mediate the export of vRNA from DMV and could also mediate the exchange of molecules between DMV and the cytoplasm ^22^. These authors also showed that once vRNA is exported, it complexes with viral N protein, association that is known to increase vRNA translation in *trans* ^36^, which could probably occur at ribosomes located at the external membrane of DMV ^16^. It is important to note that MDV have a very short shedding dynamic (~30 s), and peak at 2 h after stimulation ^26, 34^, this could explain why the shedding step is not frequently spotted by TEM. Interestingly, in a recent paper, an alternative mechanism of mitochondria fission has been described that occurs under stress and high energy demand, that depends upon the MOM molecule Fis1, which yields small mitochondria destined for mitophagy ^37^. A common feature of RO are nearby mitochondria, which may show signs of cisterna swelling and disorganization, similar to mitochondria with induced MDV shedding ^26, 27, 29, 32^, or membrane continuity with DMV ^16, 19, 23– 25^. Nevertheless, some TEM images have shown budding of what could be DMV from mitochondria ( Figure 2B). In some cases, in closely located DMV and mitochondria, vRNA signal can be observed within both, apposed to each other ( Figure 2C). Moreover, in nsp4 temperature-sensitive mutants, at a non-permissive temperature, there is an increase in mitochondria size, with enlarged cisternae, and increased localization of nsp4 and nsp3 at mitochondria, perhaps resulting from the reduction of MDV shedding, that in turn results in a reduced number of DMV ^15^. Interestingly, electron tomography has shown what could be an intermediate between DMV and MDV, a vesicle tethered to the ER with a double-membrane that contains a cisterna-like arrangement of the inner membrane ( Figure 2D) ^23^. Further support for the notion that mitochondria could be targets of SARS-CoV-2 vRNA infection that leads to DMV assembly, comes from the observation that shows mitochondria containing newly synthesized vRNA ^19^. This suggests that mitochondria somehow get vRNA that could induce stress and therefore shedding of MDV (/DMV), where the vRNA replication machinery and newly synthesized vRNA are mostly located ^19^. Interestingly, it was recently found that Fe-S cofactors, of which biosynthesis initiates at the mitochondria, are involved in SARS-CoV-2 RdRp function ^38^. Moreover, a recent *in silico* analysis predicted SARS-CoV-2 RNA localization to host mitochondria and nucleolus, further supporting this idea ^39^. In addition, it is possible that the abundance of vRNA and viral proteins within mitochondria are under tight control through the shedding of MDV, and thus, that only a few vRNA are found within mitochondria at a given moment. Interestingly, some images have shown that vRNA located inside of mitochondria is polarized towards DMV pools ( Figure 2C and E) ^19^.

Supporting also the role of mitochondria for DMV assembly is the unexpected identification of several mitochondria molecules involved in different mechanisms of its physiology (electron transport, metabolism, mitochondria ribosomes, RNA maturation, and cellular immune signaling) as interactors of viral proteins ^40^. Some of the putative relevant interactions that this work identified is that of nsp4 with the inner mitochondria membrane translocase (TIMM) complex, the interaction of ORF9b with TOMM70, and the interaction of nsp6 and ORF9c with the Sigma receptor. This receptor has been involved in several mitochondria functions, related to its location at the mitochondria-associated ER membranes (MAM) ^41^, enriched with interactors of nsp2 and 4. Additional intriguing, unexpected interactions were those of SARS CoV-2 membrane (M) protein with FASTKD5, involved in mitochondrial RNA maturation, and that of nsp8 with different mitochondria ribosomal proteins (MRP). Strikingly, interactions of ORF3a and M protein with relatives of known partners of MULAN1 (REEP and TRIM) were also identified. In a different study, nsp2 was found to interact with VDAC2 ^42^, the mitochondrial porine, whereas the mitochondria antiviral-signaling protein (MAVS) has also been identified as a target of SARS-CoV-2 infection ^10^. Together, these interactions of viral proteins with the host support that mitochondrial function is very relevant for SARS-CoV-2 infection. Furthermore, since the ribosome, mitochondrial RNA maturation and translocation mechanisms are targets of viral proteins, that according to the current model of infection are unexpected, these findings also hint that SARS-CoV-2 infects mitochondria as part of its replication cycle, rather than only taking control of this organelle through viral proteins synthesized elsewhere. The down-regulation of mtDNA encoded genes and mitochondrial RNA in patient autopsies by SARS-CoV-2 also supports this notion ^43^. Other relevant interactions of the viral proteome with mitochondrial proteins have been analyzed by others ^8, 10, 42^. A key question in this scenario is which are the steps that mediate the shedding and transformation of MDV into DMV, and how viral proteins are involved ( Figure 1 question mark 1).

## Mitochondria infection by vRNA

**Figure 3.**
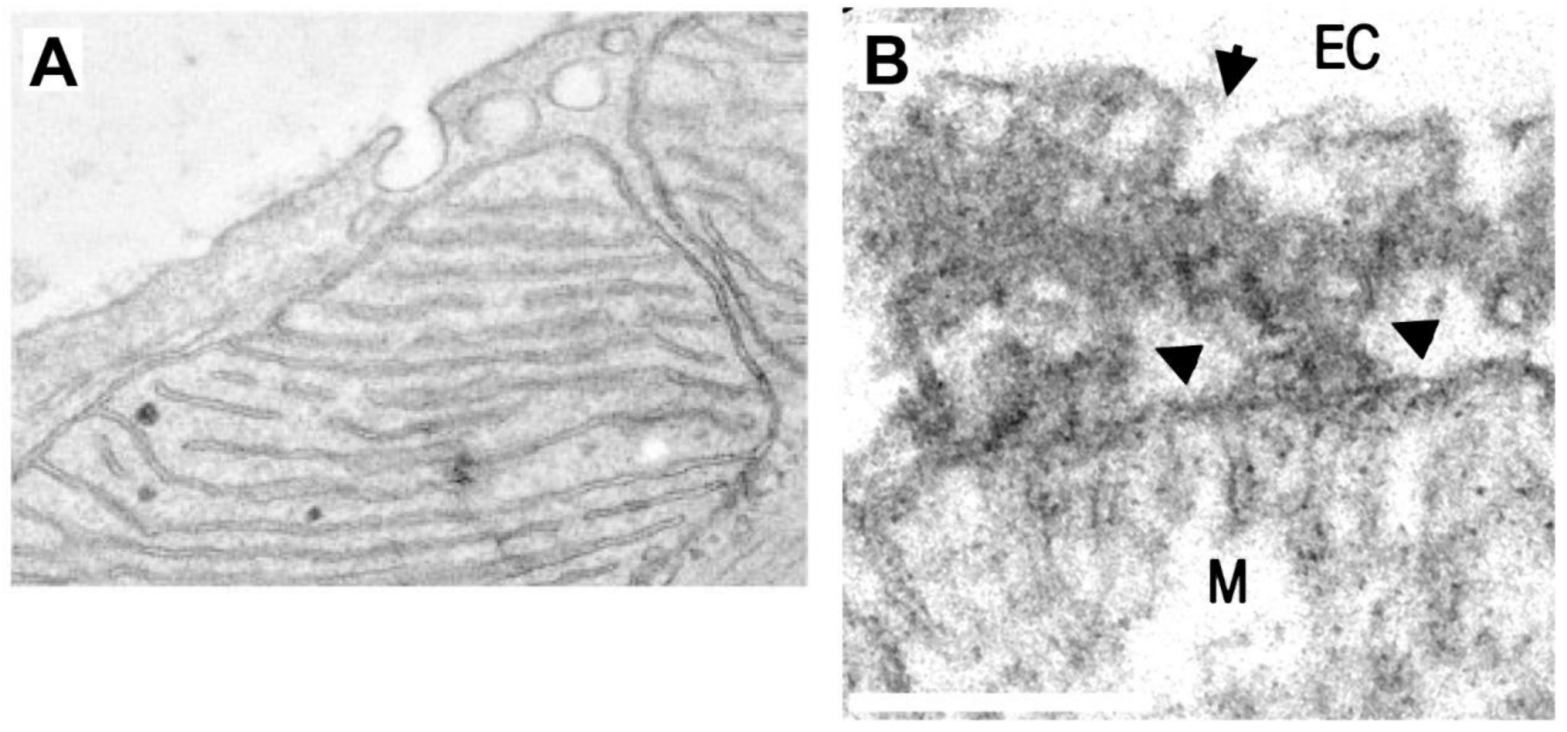
Plasma membrane-mitochondria interactions and caveolae. **A** TEM image of a cardiac myocyte in which caveolae are closely apposed to mitochondria. In this work it was found that PM–mitochondria transfer of caveolin increased cellular fitness against ischemia-reperfusion (scale not-defined) (Taken with permission from 45). **B** TEM of PM–mitochondria bridges that we recently described in cultured astrocytes. These structures consist of a highly electrodense region between PM and mitochondria (arrowheads) which is associated with vesicles with the size of caveolae (arrow), flattening of the mitochondria membrane facing the PM, and dots within mitochondria that also presents cisternae perpendicular to the PM, similar to mitochondria adherens complex (MAC) in neurons ^56^. These structures mediated the mass transfer from PM to mitochondria in minutes (bar=250 nm) (Taken with permission from 46).

How MDV–DMV are induced by SARS-CoV-2? This question has no answer yet; however, there are at least two main possibilities that are non-self-exclusive: one that is consistent with the current paradigm is that viral proteins synthesized at the ER and/or its membrane modifications in the RO somehow reach mitochondria, modulate its physiology and induce DMV, in 1–2 hours. In this regard, there are some viral proteins with mitochondria localization sequences such as 3b ^44^, or that target proteins at the inner mitochondria membrane (IMM) ( *i.e*., TIMM, electron transport proteins, and MRPS), at the MOM (TOM), or at the mitochondria matrix (FASTKD5). Alternatively, the mitochondria could start viral protein synthesis with ribosomes located at the MOM ^45^, and/or uptake vRNA from the cytoplasm after virus entry and vRNA release into the cytoplasm. In this regard, it is known that mitochondria are capable of importing RNA from the cytoplasm through a pathway that involves the TOM/TIMM complex ^46^, and SARS-CoV-2 RNA is predicted to locate at this organelle ^39^. On the other hand, a tantalizing possibility is that vRNA accesses mitochondria directly from vesicles shed from the plasma membrane (PM) in which SARS-CoV-2 is endocytosed. This PM–mitochondria pathway mediates caveolin transport to mitochondria in myocytes after stress ^47^ ( Figure 3A), and it could be an early step of what we called plasma membrane-mitochondria bridges, which we recently described in astrocytes ( Figure 3B) ^48^, involved in the emerging pathway of PM–mitochondria interactions ^49^. These PM-mitochondria bridges contain vesicles, most probably caveolae and mediate mass transfer from PM to mitochondria in minutes. Given that DMV are induced early by coronavirus (1–2 h p.i.), direct access of vRNA to mitochondria seems plausible, providing also the possibility to synthesize some viral proteins at IMM tethered mitoribosomes, with which nsp8 interacts ^40^. Notably, mitoribosomes synthesize most exclusively membrane proteins that are co-translationally inserted into the membrane with the participation of OXA1 ^50, 51^, as it is the case of transmembrane-containing nsp3, 4 and 6, involved in vRNA replication, of which nsp3 and 4 have been located at DMV and colocalize with nsp2, 5, 8, 12, 13 and 15 ^16, 19, 22, 24, 25^. Interestingly, the M protein could optimize vRNA translation through its interaction with mitochondrial FASTKD5 protein ^40^, involved in mitochondrial RNA maturation ^50^, and at the same time, viral replication could profit the mitochondria synthesized Fe-S cofactors required for RdRp function ^38^. This scenario could provide the advantage of the protected environment of mitochondria matrix, rich in ATP, avoiding the requirement of large amounts of protein to be transported from ER to mitochondria that would require energy and time.

However, the main concern against the idea that mitochondria can directly uptake SARS-CoV-2 from PM caveola comes from one study suggesting that SARS-CoV endocytosis is caveolin-independent ^52^. This finding is based on the observation that cholesterol sequestration (one of the main components of lipid rafts that in turn is endocytosed by caveolae, ^50^) with nystatin and filipin did not block pseudovirus entry. Indeed, nystatin enhanced it, whereas another cholesterol sequestering molecule, MβCD, did block it, therefore raising doubts about how cholesterol is involved. As matter of fact, different mechanisms of endocytosis have been found to mediate SARS-CoV-2 internalization ^52, 53– 55^. In addition, in this study and a different one with CoV NL63, a major lack of colocalization between viral spike protein and caveolin-1 at 20 or 60 min p.i. was also considered as evidence for a caveolin-independent mechanism. However, the putative fast dynamic nature of this interaction (since we found that mass is transferred from PM to mitochondria in ~2 min) nor extracellular conditions were considered in these approaches. Extracellular conditions are expected to be acidified in the inflammatory setting and the extracellular acidification rate (ECAR) has been found increased in SARS-CoV-2 infected monocytes ^31^. Importantly, extracellular acidification is known to induce transfer of caveolae to mitochondria ( *Reviewed in 47*). In addition, some evidence supports a role of the caveolae pathway in SARS-CoV-2 endocytosis: a) lipid rafts integrity is required for SARS-CoV entry and ACE2 is localized into lipid rafts, that are endocytosed through caveolae, well-known signaling hubs ^54, 57^; b) the S protein co-fractionates with caveolin-1 after binding to ACE2 ^54^; c) an *in silico* approach found that SARS-CoV-2 proteins S, M, orf3, and replicase 1AB have putative caveolin binding motifs ^58^; and d) orf3a protein binding to caveolin has been demonstrated experimentally ^59^. Thus, the precise role of cholesterol, caveolae, and caveolin for SARS-CoV-2 infection requires further investigation, because direct viral targeting to mitochondria could be of great relevance for SARS-CoV-2 infection. Interestingly, cholesterol-bound RNA probes are targeted to mitochondria ^60^. Furthermore, several alternative entry factors to ACE2 and facilitators capable to mediate SARS-CoV-2 infection have been identified ^61– 64^, and they could mediate SARS-CoV-2 caveolae-mediated endocytosis. Taken together, it is possible that the diversity of receptors and entry pathways exploited by SARS-CoV-2, together with the fast dynamics of PM–mitochondria communication can obscure the caveolae role.

## Conclusions

### Integration into the model of SARS-CoV-2 infection and pending questions

According to the published literature, it seems possible to conceive that SARS-CoV-2 DMV have a mitochondrial origin, through the shedding of MDV as shown in Figure 1. This possibility is supported by different observations reviewed here and would include mitochondria infection by vRNA, which could occur by its uptake from the cytoplasm through the TOM complex or direct targeting to mitochondria of PM caveolae containing SARS-CoV-2 ( Figure 1, question mark 3), as it has been reported in myocytes and astrocytes. A major advantage of the proposed role of mitochondria in DMV assembly, in comparison with their origin from the ER, is the shortest time to induce DMV, since protein synthesis required to induce ER zippering and bending would not be necessary until later when the ER is infected by vRNA. However, still many questions remain in this scenario, and most probably, previous findings that escaped this review may challenge this hypothesis, that nevertheless pretends to be an integrative starting point to further examine SARS-CoV-2 infectious cycle. For instance, is it possible that contacts between DMV and other RO-modified membranes could be related to MAM?, structures that mediate ER–mitochondria interactions and are critical for their function ^41^. It is important also to elucidate the steps that promote MDV shedding after vRNA infection and how these MDV transform into DMV ( Figure 1, question mark 1). In this regard, it is also possible that other mitochondrial molecules can be present at MDV/DMV, that increase viral fitness. Another relevant question is the origin of ribosomes that decorate DMV, that could assemble *de novo* with the action of viral proteins similar to MOM-tethered ribosomes ^45^, that are related with the PINK1/Parkin pathway, and whether they are involved in the immediate translation of vRNA after its export from DMV ( Figure 1, question mark 2). Also, the identification of FASTKD5 as an interactor of M protein opens the possibility that within mitochondria, vRNA could be target of maturation, which in turn could optimize viral protein synthesis at mitoribosomes, or when this processed vRNA are exported from DMV. In this regard, codon variation in the human mitochondria genetic code could provide clues that support or reject this hypothesis ^65^. Another intriguing possibility that should be tested is whether DMS eventually become vesicles with virions inside ( Figure 1, question mark 4). This is because, the fate of DMS is not clear, however, given the topology of their membranes and the nearby synthesis of viral proteins (perhaps in the interior of DMS), it is conceivable that the closed inner membrane becomes the viral membrane, deployed later to the ERGIC. Also pending is whether CM are the byproduct of DMV or DMS, as it has been proposed ^19, 25^. CM could be collapsed DMV that exhausted available resources in their vicinity, since CM increase after DMV formation slows down ^25^, and/or debris that remains after DMS assembly. Both mechanisms are consistent with the accumulation of viral proteins at CM. In addition, could this alternative pathway of SARS-CoV-2 cell infection be related with the lack of effect of drugs that target the endolysosomal pathway? All these questions require further research to be answered and would test this complimentary model of SARS-CoV-2 infection of mitochondria and DMV assembly. Nevertheless, it seems clear that a diversity of cellular mechanisms (entry factors and facilitators, endocytosis, cleaving proteases, organelles) are exploited by SARS-CoV-2 to infect cells, replicate and succeed.

Given the pandemic emergency worldwide, a deeper understanding of the cellular mechanisms that are exploited by SARS-CoV-2 to infect cells seems urgent as it could lead to envisage novel therapeutic targets and alternatives to control or stop the pandemic that today is still enhancing the death toll. The model proposed here for SARS-CoV-2 infection and DMV assembly provides a non-conventional scenario to explore, that could help to treat or prevent SARS-CoV-2 infection, for instance with mitochondria-targeted molecules ( *i.e.* chloramphenicol alone or in combination with other drugs; mitochondria-targeted RNA; mitochondria protein/cofactor synthesis and function), some of which have been identified as candidates to treat COVID-19 ^40^.

## Abbreviations

**CM**-convoluted membranes; **DMS**-double membrane spherules; **DMV**-double membrane vesicels; **ER**-endoplasmic reticulum; **ERGIC**-ER-Golgi intermediate compartment; **IMM**-inner mitochondria membrane; **M**-mitochondria; **MAM**-mitochondria associated membrane; **MAVS**-mitochondria antiviral-signaling protein; **MOM**-mitochondria outer membrane; nsp-non-structural protein; **nsp**-non-structural protein; **PM**-plasma membrane; **TIMM**-translocase of the mitochondrial inner membrane; **TOM**-translocase of the mitochondrial outer membrane; **VDAC**-Voltage-dependent anion channel; **VP**-vesicle packet (not shown); **vRNA**-viral RNA; **zER**-zippered ER.

## Data availability

*All data underlying the results are available as part of the article and no additional source data are required.*

## Authors' contributions

PMOB did all work related with this manuscript.
