# Evidence of mitochondria origin of SARS-CoV-2 double-membrane vesicles: a review.

**DOI:** 10.12688/f1000research.73170.3

**Published:** 2024-07-10

**Authors:** Pavel Montes de Oca-B

**Affiliations:** 1 Neurociencia Cognitiva, Instituto de Fisiologia-UNAM, CDMX, CDMX, 04510, Mexico; 2 Unidad de Neurobiologia Dinamica, Instituto Nacional de Neurologia y Neurocirugia, CDMX, CDMX, 14269, Mexico

**Keywords:** SARS CoV-2; DMV; MDV; mitochondria; caveolae; COVID

## Abstract

Coronavirus Disease-19 (COVID-19) pandemic is caused by SARS-CoV-2 that has infected more than 600 million people and killed more than 6 million people worldwide. This infection affects mainly certain groups of people that have high susceptibility to present severe COVID-19 due to comorbidities. Moreover, the long-COVID-19 comprises a series of symptoms that may remain in some patients for months after infection that further compromises their health. Thus, since this pandemic is profoundly affecting health, economy, and social life of societies, a deeper understanding of viral replication cycle could help to envisage novel therapeutic alternatives that limit or stop COVID-19.

Several findings have unexpectedly discovered that mitochondria play a critical role in SARS-CoV-2 cell infection. Indeed, it has been suggested that this organelle could be the origin of its replication niches, the double membrane vesicles (DMV). In this regard, mitochondria derived vesicles (MDV), involved in mitochondria quality control, discovered almost 15 years ago, comprise a subpopulation characterized by a double membrane. MDV shedding is induced by mitochondrial stress, and it has a fast assembly dynamic, reason that perhaps has precluded their identification in electron microscopy or tomography studies. These and other features of MDV together with recent SARS-CoV-2 protein interactome and other findings link SARS-CoV-2 to mitochondria and support that these vesicles are the precursors of SARS-CoV-2 induced DMV. In this work, the morphological, biochemical, molecular, and cellular evidence that supports this hypothesis is reviewed and integrated into the current model of SARS-CoV-2 cell infection. In this scheme, some relevant questions are raised as pending topics for research that would help in the near future to test this hypothesis. The intention of this work is to provide a novel framework that could open new possibilities to tackle SARS-CoV-2 pandemic through mitochondria and DMV targeted therapies.

## Background

Coronavirus Disease-19 (COVID-19) pandemic is caused by the +RNA coronavirus SARS-CoV-2 that has infected now more than 600 million people and has killed more than 6 million people worldwide ^1^, since it has no definitive and effective treatment until today. This infection affects mainly certain groups of people that have high susceptibility to present severe COVID-19 due to comorbidities that include cardiovascular, chronic kidney, respiratory or liver disease, severe obesity, or hypertension among others. In these patients, the cytokine storm induced by the virus poses a serious death risk due to the systemic inflammation and multiorgan failure ^2– 6^. Moreover, the so called long-COVID-19 comprises a series of symptoms that may remain in some patients for months after infection that further compromises their health, even after non-severe COVID-19 ^7^. Despite huge efforts to stop infections and deaths worldwide, only a few treatments have been demonstrated to ameliorate severe COVID-19, and different vaccine strategies are currently under investigation in clinical phase IV trials. Unfortunately, it is now clear that the intended vaccine objective, the prevention of COVID-19 ^8^, was not fulfilled adequately due to waning immunity ^9^. Therefore, in this scenario, a deeper understanding of the cellular mechanisms exploited by SARS-CoV-2 for cell infection and replication could undoubtedly provide new unforeseen strategies to tackle this pandemic.

## The SARS-CoV-2 Replication Organelles (RO) and the unresolved origin of DMV

Several recent reports have shown that mitochondria play a relevant role during SARS-CoV-2 infection ^10– 14^. These findings and previously published results of SARS-CoV-2, SARS-CoV and other coronavirus biology let hypothesize that mitochondria could be responsible for the assembly of double-membrane vesicles (DMV). These are membrane modifications induced by SARS-CoV-2 where virus replication occurs in the infected cell, assumed to protect vRNA from the intracellular environment and prevent the antiviral response. Currently, DMV are mainly believed to be derived from the endoplasmic reticulum (ER) or other mechanisms as autophagy ^15– 17^. However, published literature supports that double-membrane mitochondria-derived vesicles (MDV), discovered almost 15 years ago ^18^, could be the precursors or relatives of DMV. This hypothesis of mitochondria role in DMV assembly and the involvement of MDV has been suggested previously ^10, 19, 20^. Indeed, mitochondria is a well-known target of different viral infections ^21^. Furthermore, specialized replication organelles (RO) at mitochondria outer membrane (MOM) have been observed in FHV ^22^, whereas HIV RNA is known to locate at mitochondria ^23^. Here, a review of the evidence that supports this notion for SARS-CoV-2 is presented and integrated into the current model ^24^, with the intention to provide a novel framework that could open possibilities to tackle the SARS-CoV-2 pandemic and COVID-19.

**Figure 1.**
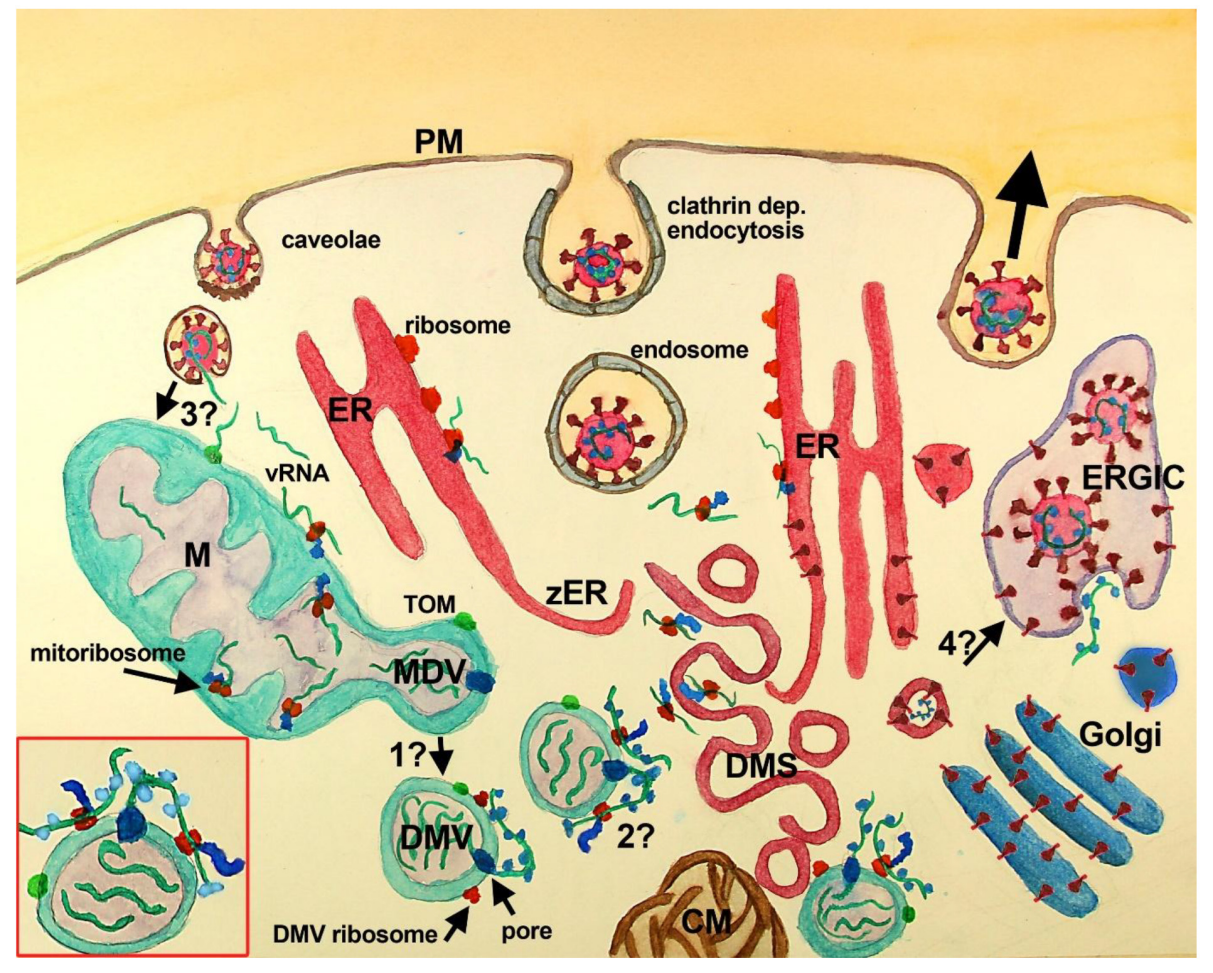
Scheme of SARS-CoV-2 replication cycle with mitochondria and MDV participation. SARS-CoV-2 recognizes ACE2 (not shown) at the PM of the host cell inducing its endocytosis. Several other entry factors and facilitators have been found to mediate SARS-CoV-2 entry into the host cells. Clathrin-dependent and independent endocytosis mediate viral entry. **In the current paradigm**, clathrin-mediated endocytosis (middle vesicle at PM) follows the endosomal pathway, that through endosomal acidification and cleavage of S protein (by TMPRSS2 or cathepsin) induces the fusion of SARS-CoV-2 membrane with vesicle membrane deploying vRNA (green stripes) into the cytoplasm. Once released, vRNA reaches cytoplasmic and ER ribosomes starting viral protein synthesis. Viral proteins induce the assembly of RO elements that are interconnected (not shown), initially inducing zER that through bending and scission assemble DMS, CM, and DMV, where synthesis of viral proteins and vRNA takes place. Proteins synthesized at ER, DMS and CM reach the Golgi, where they are posttranslationally modified, and the ERGIC, where the viral particles are assembled and set ready for exocytosis (large black arrow, right vesicle at PM). Viral particles have also been observed at multivesicular bodies (not shown). In this paradigm, DMV are believed to derive from DMS and/or CM, although some controversies have been raised (see text), mainly the temporal sequence, the lack of ER markers in DMV and the lack of intermediate structures. VP (not shown) are formed by the fusion of single DMS. In this paradigm, the mitochondrial role is not considered, although evidence of its participation has accumulated (see text). **In the complementary scenario** proposed in this work, DMV are shedded from mitochondria, through a mechanism similar to that described for double-membrane MDV, and or asymmetric mitochondria fission, both known to be potentiated after mitochondrial stress. Whether MDV require several transforming steps to become DMV, or if these compartments are essentially the same with viral proteins included needs further investigation ( **question mark 1**). DMV have double-membrane spanning pores (dark blue) in which nsp3 is inserted along with other unidentified molecules. These pores mediate the export of vRNA to the cytoplasm, which complexes with N protein outside DMV. Exported vRNA may be translated immediately by ribosomes located in the external membrane of DMV ( **question mark 2**). Interestingly, some MDV have been shown to carry mitochondrial proteins from the IMM, MOM, and mitochondria matrix, which could be also present at DMV (green dot at MOM, MDV/DMV outer membrane). In this complimentary scenario, a critical question is how vRNA accesses mitochondria ( **question mark 3**). It is possible that vRNA once in the cytoplasm is translated at MOM ribosomes. In the last months, while this manuscript was being reviewed, vRNA has been demonstrated within mitochondria, location that depends upon TOM function ^34, 35^. vRNA could reach mitochondria through the fusion of caveolae with endocytosed SARS-CoV-2, although it is not clear yet whether coronavirus can follow this pathway. Finally, an intriguing possibility, that could be critical, is whether DMS, which are induced by viral proteins most probably synthesized nearby the ER from where they are derived, may transform into vesicles with viral particles. This possibility is supported by the synthesis of viral proteins at DMS and would require that vRNA is packed inside, which seems feasible because protein synthesis at DMS implicates the presence of vRNA. The lack of *de novo* synthesized vRNA at DMS indicates that vRNA synthesis does not happen there, but does not rule out the presence of vRNA within DMS. **Inset, red square left bottom:** A close-up of a DMV with vRNA in its interior (3 green stripes), with one vRNA being exported by the double-membrane spanning pore (dark blue at DMV membranes), complexing with the N protein (light blue). This exported vRNA could be translated in situ at DMV ribosomes (red at DMV external membrane). Peptides synthesized by DMV ribosomes are shown attached in blue and the green dot at DMV represents mitochondrial molecules located at DMV. **Abbreviations**: **CM**-convoluted membranes; **DMS**-double-membrane spherules; **DMV**-double-membrane vesicles; **ER**-endoplasmic reticulum; **ERGIC**-ER-Golgi intermediate compartment; **M**-mitochondria; **PM**-plasma membrane; **TOM**-translocase of the mitochondrial outer membrane; **VP**-vesicle packet (not shown); **vRNA**-viral RNA; **zER**-zippered ER.

DMV along with other membrane modifications are part of the RO induced by SARS-CoV-2. Other membrane modifications include convoluted membranes (CM), zippered ER (zER), vesicle packets (VP), and double-membrane spherules (DMS) ^24^ ( Figure 1). RO are induced by SARS-CoV-2 in infected cells, as a variety of RO are induced by viruses including other nidovirus and picornavirus among others ^15, 17^. DMV assembly is induced by viral proteins but seems to also require other viral or host factors because cell plasmid transfection of transmembrane-containing non-structural proteins (nsp) 3, 4, and 6 induced membrane arrangements that resemble DMV but with a smaller size ^25^. These nsp are part of the complex involved in viral RNA (vRNA) replication, together with nsp12, the RNA-dependent RNA polymerase (RdRp), and other nsp. Consistently, nsp4 mutation alters the assembly of DMVs ^19^ and abolishes viral replication ^26^. Nsp4, 3, and the nuclear (N) protein are located at DMV ^20, 24^, where nsp3 has been recently shown to form a pore complex, elusive for some time, that communicates DMV interior with the cellular cytoplasm, complex that could also involve host factors and/or other viral proteins ^27^. Surprisingly, it has been shown that RNA synthesis in SARS-CoV infected cells responds differentially to two different inhibitors of protein translation. Cycloheximide, that inhibits eukaryotic translation partially prevented RNA synthesis, whereas puromycin, that inhibits eukaryotic and prokaryotic translation almost fully blocked RNA synthesis ^28^. This finding suggests that the synthesis of proteins relevant for vRNA replication occurs at the mitochondria.

Most membrane modifications induced by SARS-CoV-2 are presumably derived from ER membranes and are interconnected by membrane contacts forming a reticulo-vesicular network ^24, 29^. In the case of DMV, the ER origin was in part assumed because such mechanism is presumed to mediate RO assembly in other +RNA viruses ^30^; because they have contacts with other membrane modifications of the RO and the ER; because ribosomes have been observed on DMV surface, and because DMS and CM were proposed to be precursors of DMV ^15, 20, 24, 25, 30^. DMV size ranges from 150 to 300 nm, but they grow through infection, and it has been well-established that SARS-CoV-2 DMV are the location where vRNA synthesis occurs ^20, 24, 31^. Moreover, RNA filaments have been visualized within DMV ^32^. Importantly, DMV are early (1–2 h post-infection [p.i.]) observed in the cell cytoplasm after coronavirus infection , and their number increase through time reaching a maximum in 6–8 h.p.i. ^20, 33^. There are currently two models for DMV assembly from the ER. In the case of coronavirus, DMV are thought to be assembled from zER that folds and closes in response to vRNA, as observed with IBV ^15, 30^, with CM and DMS as putative intermediate precursors ^20, 24^. However, major challenges remain for this model, because no intermediate structures have been recognized between DMV and DMS or CM, and because CM and DMS, both derived from the ER, have no relationship with DMV beyond their membrane contacts ^20, 24^. Moreover, DMV do not have ER (nor ERGIC, Golgi, or endosomal/lysosomal) conventional markers as it would be expected if they were assembled from the ER. Only partial overlap of PDI or colocalization of non-conventional markers have been reported (calnexin, reticulon and sec61), but interestingly, these have been identified as part of the proteome of ER-mitochondria contacts, also called mitochondria associated membranes (MAMs) ^29, 33, 36– 40^. Most importantly, it has been shown that DMV assembly (1-2 h p.i.) precedes CM assembly (~3h p.i.) ^33^. Furthermore, CM and DMS do not carry out vRNA synthesis as DMV do ^24^. Thus, CM and DMS seem not to be DMV precursors ^24^. On the other hand, the high energetic and complex topological requirements assumed to occur for DMV assembly through the zippered ER model, given their numbers after a few h.p.i, further complicate this notion. Indeed, it has been suggested that DMV could have another origin than the ER ^10, 15, 24^.

## The mitochondria and double-membrane MDV as the putative origin of SARS-CoV-2 DMV

**Figure 2.**
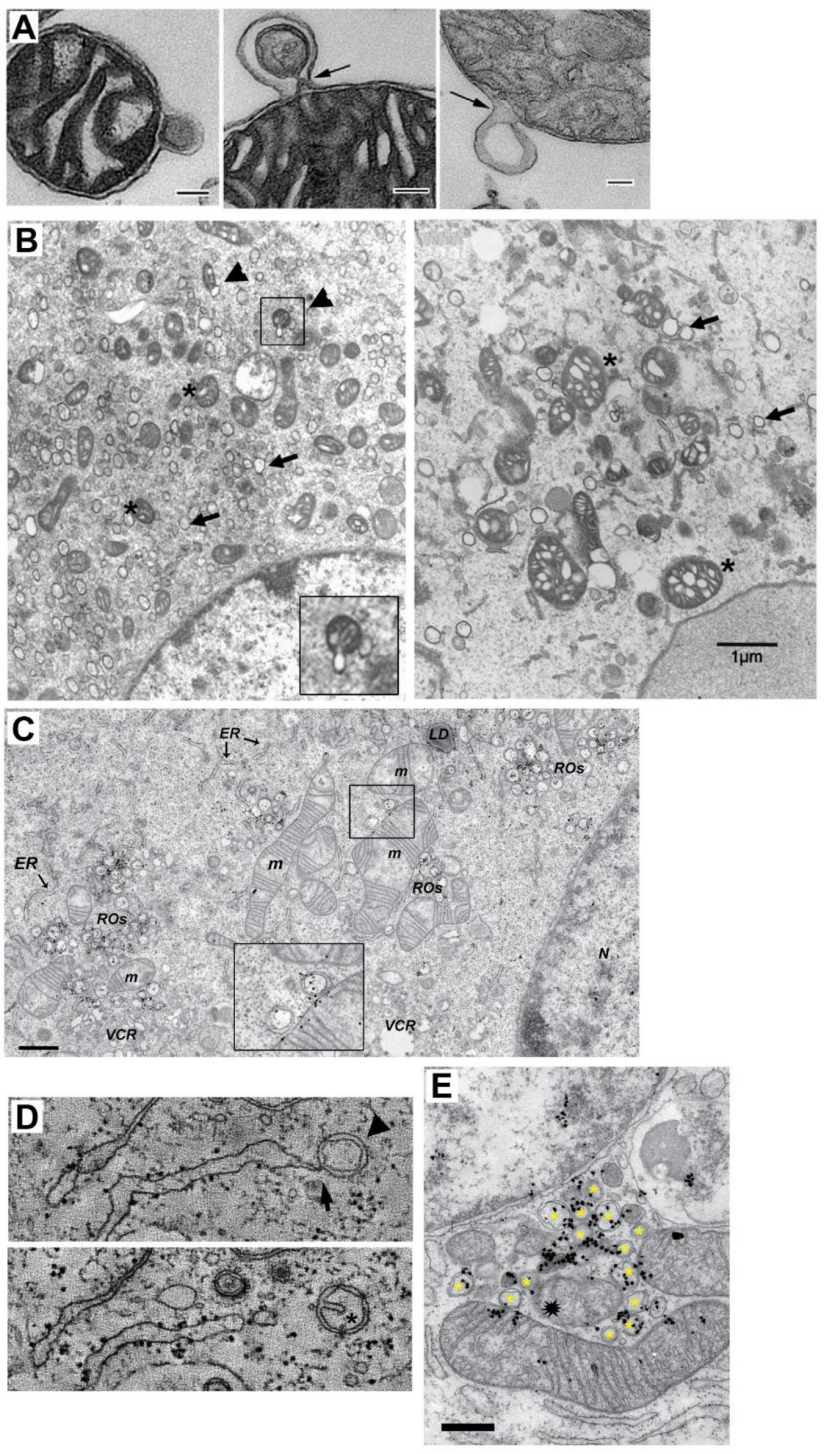
Double membrane MDV and DMV-mitochondria relationship. **A** TEM of three isolated mitochondria from bovine heart shedding double membrane vesicles (bar=500 nm in first panel and =100 nm in panels 2 and 3). ( *Taken from 45*). **B** TEM analysis of mitochondria morphology and DMV formation in infectious clone virus (icv)-infected cells (derived from coronavirus murine hepatitis virus; MHV). This icv has a mutated nsp4-N258T that results in temperature sensitive viral replication. As observed, after 5.5 h.p.i at the permissive temperature, DMV are evident (arrows) and some mitochondria (asterisk) are associated with shedded vesicles (left panel, arrowheads and inset). Interestingly, if cells are left at the non-permissive temperature the last 2 h. (right panel), mitochondria appear swollen with enlarged cisternae, and accompanied by increased localization of nsp3 and nsp4 at mitochondria (not shown). ( *Modified from 19 with permission*) (bar=1000 nm). **C** TEM and autoradiography of *de novo* synthesized vRNA in SARS CoV infected-Vero E6 cells at 7 h.p.i. As it is observed, some DMV are closely located to mitochondria, showing both vRNA label within. Interestingly, vRNA label in DMV and mitochondria are neighboring (inset) ( *Modified from 24*). **D** Electron tomography images of an Infectious Bronchitis Virus-infected cell showing a DMV (arrowhead) connected to the ER (arrow) (upper panel). In a different plane (lower panel), the same DMV shows a cisternae-like arrangement of the inner membrane (asterisk), that resembles those of mitochondria, hinting the putative mitochondrial origin of DMV (Modified from *30* with permission). **E** TEM and autoradiography of *de novo* synthesized vRNA in SARS CoV infected-Vero E6 cells at 12 h.p.i. It can be observed that DMV (asterisks) are densely labeled with vRNA signal. Notably, a couple of mitochondria are also labeled for vRNA, but importantly, this label is polarized in both mitochondria near the membrane towards the DMV cluster. Intriguilgly, within this pool of DMV, a degenerated-like mitochondria structure is observed (spark at the center), perhaps resulting from extensive DMV shedding (bar=500 nm) (Modified from *24*).

Given the recent findings that relate SARS-CoV-2 with mitochondria, it is possible that MDV are the origin of DMV. MDV were discovered almost 15 years ago, and they comprise different kind of vesicles shed from mitochondria with selective cargo and markers (TOM+ or MAPL+). They are involved in intracellular traffic to different organelles and mitochondria quality control ^18, 41– 44^. Interestingly, some TOM+ MDV have a double-membrane with an average size of 160 nm (range 80–250 nm) ( Figure 2A) ^43^. Initially, MDV were found to be shed independently of drp1, mitochondria fission, and autophagy, however, a more recent analysis with improved tools has shown that drp1 is essential for their scission ^41, 43, 45^. Several coronavirus proteins have been shown to down- or up-regulate drp1 (N, envelope [E], nsp3, nsp4a, nsp4b, and orf9b), molecule that is critical for mitochondria fission ^12^. In particular, SARS-CoV orf9b expression results in drp1 degradation through the proteasomal pathway, casting doubts about the role of MDV as DMV precursors or relatives. However, it must be noted that this evidence was gathered with a cell line stably transfected with orf9b, thus, its expression kinetics profile does not correspond to that of an actual viral infection ^46^. Indeed, in an infected cell, the expression of orf9b could concur with DMV generation decrease.

MDV are generated in steady conditions and have been observed *in vivo* ^47^, but their number increases after mitochondrial stress or higher respiratory activity ^41, 45^. In this regard, it has been found that SARS-CoV-2 infected alveolar epithelial cells, monocytes and PBMC have compromised mitochondrial function and energy deficit ^48– 51^, even months after infection clearance and symptoms relief ^52^. These observations could be the long-term result of viral infection in which mitochondria shedding of MDV/DMV, triggered initially by a burst of metabolic activity and/or stress, leads to mitochondria function impairment. In this regard, OXPHOS impairment, ROS increase and HIF1α activation, that reprograms cells towards glycolysis, have been reported after viral infection ^34, 50, 53^. ROS stress could be the trigger to induce MDV/DMV production, although this needs to be tested since nsp 3, 4 and 6 transfection induce the generation of DMV-like vesicles ^25^. Furthermore, mitochondria phenotype alteration has been observed *in vitro* ( Figure 2E) ^24, 34, 48^ and *in* COVID-19 postmortem specimens ^54^ that may comprise fusion, reported in most infected cells, or fission which has also been observed ^50, 54^. On the other hand, mitochondria disturbance in infected patients could also be due to angiotensinII excess, that results after ACE2 downregulation by SARS-CoV infection or Spike (S) protein binding ^55– 59^.

MDV not only look like DMV for their double-membrane and approximate size, they also transport selective cargo to peroxisomes or the endolysosomal system after mitochondrial stress, depending on Vps35 and syntaxin-17/SNAP29/VAMP7, respectively ^41, 45, 60, 61^. Indeed, it has been recently reported that MDV may compensate for the loss of mitophagy, thus keeping mitochondria quality control, an in SARS-CoV-2 infected cells mitophagy is impaired ^34, 44^. The intracellular traffic of MDV offers a pathway that could be involved in the intracellular transport of viral components to secondary vesicular structures (i.e. ERGIC, lysosomes, multivesicular bodies) where viral particles are assembled and set ready for exocytosis. Indeed, mitochondrial proteins have been identified in exosomes from SARS-CoV-2 infected neurons and astrocytes ^62^. Interestingly, PINK1/Parkin and the mitochondrial ubiquitin ligase MULAN1 (MAPL) have been involved in MDV shedding from mitochondria ^45, 63^ and it is known to be involved in the antiviral response of mitochondria ^64^.

Proteomic studies have shown that MDV may contain 100–200 different mitochondrial molecules, including subunits of the translocase complexes of the MOM (TOM 20, TOM70, TOM40) and inner mitochondria membrane (IMM) (TIMM22, TIMM23 and TIMM29); components of the OXPHOS complexes (COX1, CYCS, UQCRC1); the VDAC1-3; enzymes of the TCA cycle; enzymes such as pyruvate dehydrogenase, lactate dehydrogenase and hexokinase; the MCU; the ATP/ADP translocase; Fe-S cluster binding proteins; and Miro1/2 among others ^41– 43, 65^. Thus, mitochondrial components and resources useful for vRNA replication are transported or could be generated within MDV, since they are “chunks” of mitochondria. It was recently shown that DMV have pores that span the double-membrane that mediate the export of vRNA from DMV and could also mediate the exchange of molecules between DMV and the cytoplasm ^27^. These authors also showed that once vRNA is exported, it complexes with viral N protein, association that is known to increase vRNA translation in *trans* ^66^, which could probably occur at ribosomes located at the external membrane of DMV ^20^. It is important to note that MDV have a very short shedding dynamic (10–30 s), and peak at 2 h after stimulation ^41, 43, 63, 67^. This could explain why the shedding step is not frequently spotted by TEM. 

TOM+ MDV have been observed to originate from mitochondrial protrusions termed mitochondria nanotubes or mitochondria derived tubules ^43, 67^. Interestingly, it has been shown that such nanotubes emerge preferentially at ER-mitochondria contacts or MAM´s, that are enriched with Miro1, critical for MDV genesis ^43, 68^. These observations open the possibility that the well-described interconnections between DMV and the ER are actually the reminiscence of these MAMs, rather than the common ER membrane shared by DMV, from where, according to the current paradigm, DMV originate ^15– 17^. These findings also indicate that MAMs could play a critical role in DMV genesis and function through their roles in Ca2+ and lipid exchange, autophagy, mitochondrial biogenesis and ER stress response ^36, 69^. Intriguingly, mitochondrial nanotubes are relevant for mDNA transfer between mitochondria, with a central role played by Mic60 that is present in MDV, however, MDV are devoid of mDNA ^43, 68^. As mentioned above, MDV shedding requires drp1 for their scission that may be recruited by adaptors MID49, MID51 or MFF, that associate with Miro1/2, that in turn mediate the formation of mitochondria derived tubules through Mic60 interaction with kinesin that pulls from microtubules ^43, 68^. Importantly, these adaptors may interact with the TOM complex through intermediate players, thus suggesting that TOM may play a pivotal role in MDV genesis. These intermediate players are endophilin B for MID49 and Huntingtin for MFF ^70^. Interestingly, the interaction between MFF and TOM22 with AQP6 has also been reported ^70^. The putative role of these molecules requires to be tested experimentally.

A common feature of SARS-CoV-2 RO are nearby mitochondria, which may show signs of cisterna swelling and disorganization, similar to mitochondria with induced MDV shedding ^41, 45, 60, 65^, or membrane continuity with DMV ^20, 24, 30– 33^. Nevertheless, some TEM images have shown budding of what could be DMV from mitochondria ( Figure 2B) ^19^. In that work with nsp4 temperature-sensitive mutants, at the non-permissive temperature, there was an increase of mitochondria size, with enlarged cisternae, and increased localization of nsp4 and nsp3 at mitochondria, perhaps resulting from the reduction of MDV shedding, that in turn resulted in a reduced number of DMV ( Figure 2B) ^19^. In some cases, in closely located DMV and mitochondria, *de novo* synthesized vRNA signal can be observed within both, apposed to each other ( Figure 2C) ^24^. Although the mathematical method employed by these authors to evaluate these labels did not identify it as positive compared with the majoritarian label observed in DMV, it is possible that the abundance of vRNA and viral proteins within mitochondria are under tight control through the shedding of MDV/DMV, and thus, that only few vRNA are found within mitochondria at a given moment. Notably, electron tomography has shown what could be an intermediate between MDV and DMV, a vesicle tethered to the ER with a double-membrane that contains a cisterna-like arrangement of the inner membrane ( Figure 2D) ^30^. Strikingly, it was recently found that Fe-S cofactors, of which biosynthesis initiates at the mitochondria, are involved in SARS CoV-2 RdRp function ^71^, and some proteins related with these clusters are present and can be enriched in MDV after oxidative stress ^42^. Moreover, a recent *in silico* analysis predicted SARS-CoV-2 RNA localization to host mitochondria and nucleolus, further supporting this idea ^72^. Indeed, this work led many to consider mitochondria infection by vRNA. As a matter of fact, while this manuscript was under review, this year two different groups demonstrated vRNA localization at mitochondria ^34, 35^. Furthermore, some images have shown that vRNA located inside of mitochondria are polarized towards DMV pools ( Figure 2C and E). In Figure 2C, it is also possible to see a degenerated-like mitochondria among DMV and mitochondria (spark in Figure 2E), probably resulting from large MDV/DMV shedding ^24^. Notably, it has been shown that SARS-CoV-2 isolated from infected individuals is capable to replicate in bacterial cultures ^73^, further endorsing that mitochondria plays a critical role for SARS-CoV-2 cycle.

Supporting also the role of mitochondria for DMV assembly is the unexpected identification of several mitochondria molecules involved in different mechanisms of its physiology (electron transport, metabolism, mitochondria ribosomes, RNA maturation, and cellular immune signaling) as interactors of viral proteins ^74^. Although most of these interactions still require to be functionally validated, some of the putative relevant interactions that this work identified is that of nsp4 with the inner mitochondria membrane translocase (TIMM) complex, the interaction of orf9b with TOMM70, and the interaction of nsp6 and orf 9c with the Sigma receptor. Of these, the TOM70-orf9b and SigmaR-Nsp6 have been validated experimentally ^75, 76^. The Sigma receptor has been involved in several mitochondria functions, related to its location at the MAMs ^69^, enriched with interactors of nsp2 and 4 ^77^. Additional, intriguing, unexpected interactions were that of SARS CoV-2 membrane (M) protein with FASTKD5, involved in mitochondrial RNA maturation, and that of nsp8 with different mitochondria ribosomal proteins (MRP). Also, interactions of orf3a and M protein with relatives of known partners of MULAN1 (REEP and TRIM) were also identified, however, given the diversity of these molecules, more work is required to test the significance of these interactions. Importantly, the orf3a protein has been reported to induce apoptosis of infected cells through the extrinsic pathway ^78^. In a different study, nsp2 was found to interact with VDAC2 ^77^, the mitochondrial porine, whereas the mitochondria antiviral-signaling protein (MAVS) has also been identified as a target of SARS-CoV-2 infection ^12^. Together, these interactions of viral proteins with the host support that mitochondrial function is very relevant for SARS-CoV-2 infection. Furthermore, since the mitoribosome, the mitochondrial RNA maturation and translocation mechanisms are targets of viral proteins, that according to the current model of infection are unexpected, these findings also hint that SARS-CoV-2 infects mitochondria as part of its replication cycle, rather than only hijacking this organelle through viral proteins synthesized elsewhere. The down-regulation of mtDNA encoded genes and mitochondrial RNA in patient autopsies and disregulation in infected cells by SARS-CoV-2 also supports this notion ^49, 50, 79^. Other relevant interactions of the viral proteome with mitochondrial proteins have been analyzed by others ^10, 12, 77^. A key question in this scenario is which are the steps that mediate the shedding and transformation of MDV into DMV, and how viral proteins are involved ( Figure 1 question mark 1).

Interestingly, in a recent paper, an alternative mechanism of mitochondria fission has been described that occurs under stress and high energy demand, that depends upon the MOM molecule Fis1, which yields small mitochondria destined for mitophagy ^80^. This could represent a different pathway involved in DMV genesis.

As explained above, DMV origin has also been proposed to be related with the autophagic pathway. However, several lines of evidence indicate that autophagy is unlikely to play a role in the generation of coronavirus replicative structures ^81^. Since MDV genesis is independent of FUDCN1, an activator of mitophagy, this also advocates against the autophagic origin of MDV/DMV ^43^. Moreover, it has been reported that SARSCoV2 infection induces and inhibits autophagy and impairs mitophagy of infected cells ^34, 82^. This compromises quality control mechanisms, further supporting MDV/DMV relationship, because it has been shown that MDV may compensate loss of mitophagy thus maintaining mitochondria quality control ^44^. Indeed, it has been suggested that autophagy modulators could treat COVID-19 given the impairment of the pathway ^82^.

Taken together, these observations strongly suggest that mitochondria could be targets of SARS-CoV-2 vRNA infection, leading to MDV/DMV assembly. Also, they indicate that mitochondria somehow get vRNA that could induce stress and therefore shedding of MDV/DMV, where the vRNA replication machinery and newly synthesized vRNA are mostly located ^24^. Some of these possibilities have already been confirmed because two different groups demonstrated this year the localization of vRNA within mitochondria of infected cells *in vitro* and *in vivo* ^34, 35^, involving TOM20 as mediator, thus substantiating the mitochondrial origin of DMV.

## Mitochondria infection by vRNA

**Figure 3.**
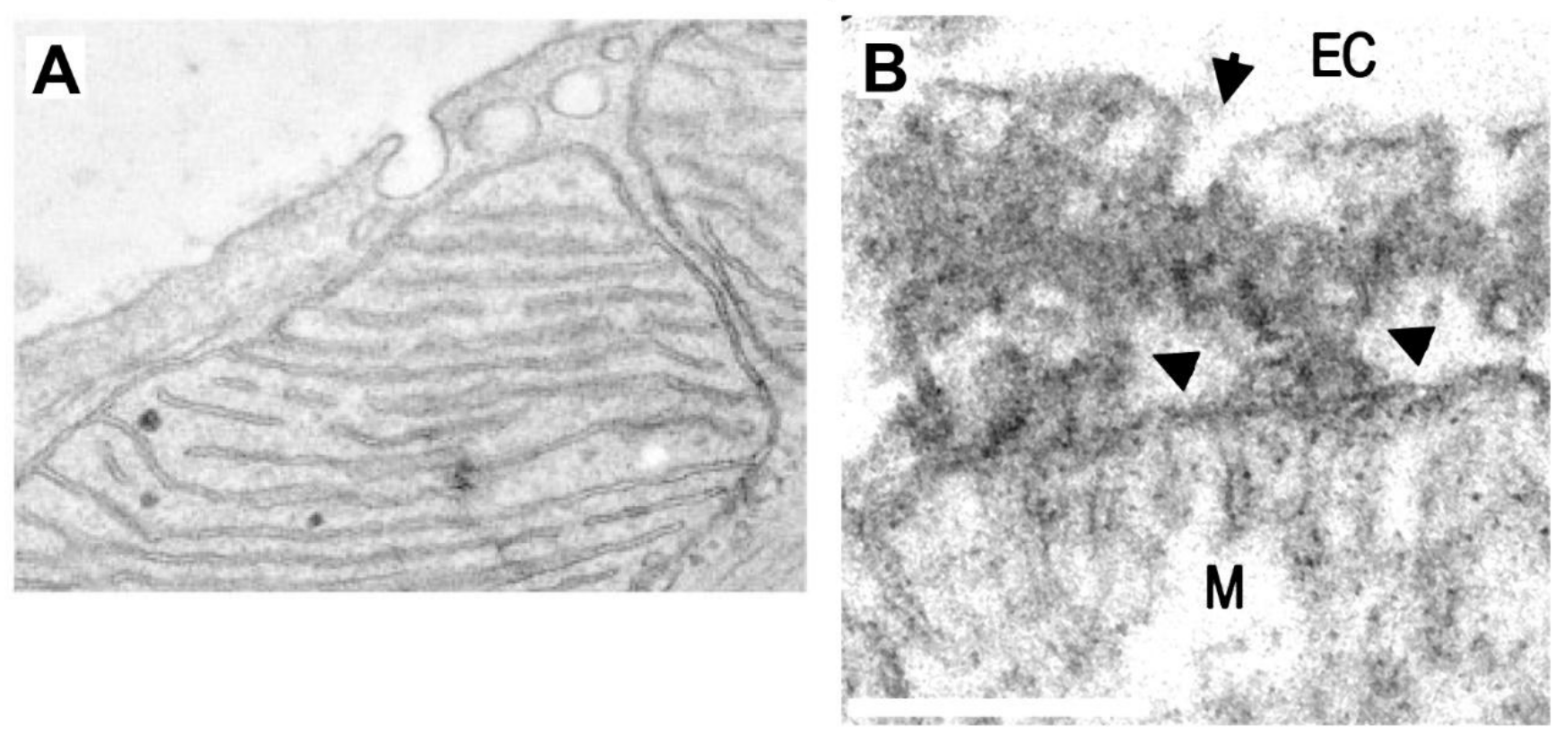
Plasma membrane-mitochondria interactions and caveolae. **A** TEM image of a cardiac myocyte in which caveolae are closely apposed to mitochondria. In this work it was found that PM-mitochondria transfer of caveolin increased cellular fitness against ischemia-reperfusion (scale not-defined) ( *Taken with permission from 86*). **B** TEM of PM-mitochondria bridges that we recently described in cultured astrocytes. These structures consist of a highly electrodense region between PM and mitochondria (arrowheads) which is associated with vesicles with the size of caveolae (arrow), flattening of the mitochondria membrane facing the PM, and dots within mitochondria that also presents cisternae perpendicular to the PM, similar to mitochondria adherens complex (MAC) in neurons ^93^. These structures mediated the mass transfer from PM to mitochondria in minutes (bar=250 nm) *(Taken with permission from 87*).

But then, how MDV/DMV are induced by SARS-CoV-2? There are at least two main possibilities that are non-self-exclusive: one that is consistent with the current paradigm is that viral proteins synthesized at the ER and/or its membrane modifications in the RO somehow reach mitochondria, modulate its physiology and induce DMV, in 1–2 hours. In this regard, there are some viral proteins with mitochondria localization sequences of SARS-CoV such as 3b ^83^, or that target proteins at the IMM (i.e TIMM, electron transport proteins, and MRPS), the MOM (TOM, VDAC), or the mitochondria matrix (FASTKD5) ^74^. Alternatively, the mitochondria could itself start viral protein synthesis with ribosomes located at the MOM ^84^, and/or uptake vRNA from the cytoplasm after virus entry and vRNA release into the cytoplasm, although the former would require mitochondria localization sequences in those translocated molecules. In this regard, it is known that mitochondria are capable to import RNA from the cytoplasm through a pathway that involves the TOM/TIMM complex ^85^, and SARS-CoV-2 RNA is predicted to locate at this organelle ^72^. Indeed, this has been confirmed in a recent work published during the review of this manuscript that demonstrated that TOM20 mediates vRNA entry into mitochondria and that it is not colocalized with ER and lysosomal markers ^34^. In addition, a tantalizing possibility is that vRNA accesses mitochondria through TOM complex directly from vesicles shedded from the plasma membrane (PM) in which SARS-CoV-2 is endocytosed. This PM-mitochondria pathway mediates caveolin transport to mitochondria in myocytes after stress ( Figure 3A) ^86^, and it could be an early step of what we called plasma membrane-mitochondria bridges, which we recently described in astrocytes ( Figure 3B) ^87^, involved in the emerging pathway of PM-mitochondria interactions ^88^. These PM-mitochondria bridges contain vesicles, most probably caveolae, and mediate mass transfer from PM to mitochondria in minutes ^87^. Interestingly, it has been observed through TEM in infected cells from patients that virus containing vesicles may reach very close to MOM, vesicles that are shedded from plasma membrane regions where abundant caveolae-like invaginations are observed, presumably containing viral particles ^54^. Consistent with this idea, it has been reported that ACE2 can be located at the mitochondria ^89^, as it has been reported for several other plasma membrane molecules ^88^, whether ACE2 reaches mitochondria from plasma membrane was suggested although not established in this work. Moreover, ACE2 is known to promote mitochondria function ^90^. These observations endorse the possibility that SARS-CoV-2 may reach mitochondria directly after its endocytosis bound to ACE2.

Given that DMV are early induced by coronavirus (1-2 h.p.i.), fast and direct access of vRNA to mitochondria seems plausible and advantageous, compared with the ER origin of DMV. This scenario also provides the possibility to synthesize some viral proteins at IMM tethered mitoribosomes, with which nsp8 presumably may interact ^74^. Notably, mitoribosomes synthesize most exclusively membrane proteins that are co-translationally inserted into the membrane with the participation of OXA1 ^91, 92^, as it is the case of transmembrane-containing nsp3, 4 and 6, involved in vRNA replication, of which nsp3 and 4 have been located at DMV and colocalize with nsp2, 5, 8, 12, 13 and 15 ^20, 24, 27, 31, 33^. Interestingly, OXA1 has been identified at MDV by a proteomic study ^42, 43^, thus supporting the idea that synthesis of proteins with transmembrane domains coded by the vRNA may occur within mitochondria and/or MDV/DMV. In addition, the M protein could optimize vRNA translation through its interaction with mitochondrial FASTKD5 protein ^74^, involved in mitochondrial RNA maturation ^91^, and at the same time, viral replication could profit the mitochondria synthesized Fe-S cofactors required for RdRp function ^71^. This scenario could provide the advantage of the protected environment of mitochondria matrix, rich in ATP, at least prior to OXPHOS reduction after infection ^50, 53^, avoiding the requirement of large amounts of protein to be transported from ER to mitochondria that would require considerable amounts of energy and time.

However, the main concern against the idea that mitochondria can directly uptake SARS-CoV-2 from PM caveolae comes from one study suggesting that SARS-CoV endocytosis is caveolin-independent ^94^. This finding is based on the observation that cholesterol sequestration (one of the main components of lipid rafts that in turn are endocytosed by caveolae, *^34^*) with nystatin and filipin did not block pseudovirus entry. Indeed, nystatin enhanced it, whereas another cholesterol sequestering molecule, MβCD, did block it, therefore raising doubts about how cholesterol is involved. As matter of fact, different mechanisms of endocytosis have been found to mediate SARS-CoV-2 internalization ^94– 97^. In addition, in that study and a different one with CoV NL63, a major lack of colocalization between viral S protein and caveolin-1 at 20 or 60 m.p.i. was also considered as evidence for a caveolin-independent mechanism. However, the fast dynamic nature of this interaction (since we found that mass is transferred from PM to mitochondria in ~2 min ^87^) nor the extracellular conditions were considered in these approaches. In this regard, it is relevant to note that extracellular acidification is known to induce transfer of caveolae to mitochondria (Reviewed in 88). Consistently, during SARS-CoV-2 infection extracellular conditions are acidified in the inflammatory setting and by the increase of the extracellular acidification rate (ECAR), related with lactate production by glycolysis reprogramming that has been found in SARS CoV-2 infected cells ^48, 98^. Moreover, acidosis has been reported in COVID-19 patients ^98^.

On the other hand, there is some evidence supporting the role of the caveolae pathway in SARS-CoV-2 endocytosis: a) lipid rafts integrity is required for SARS-CoV entry and ACE2 is localized into lipid rafts, that are endocytosed through caveolae, well-known signaling hubs ^96, 99^; b) the S protein co-fractionates with caveolin-1 after binding to ACE2 ^96^; c) an *in silico* approach found that SARS-CoV-2 proteins S, M, orf3, and replicase 1AB have putative caveolin binding motifs ^100^; d) orf3a protein binding to caveolin has been demonstrated experimentally ^101^; and e) cholesterol has been shown to enhance cellular infection though ACE2 endocytosis ^102^. Thus, the precise role of cholesterol, caveolae, and caveolin for SARS-CoV-2 infection requires further investigation, because direct and fast viral targeting to mitochondria could be of great relevance for SARS-CoV-2 infection. Interestingly, cholesterol-bound RNA probes are targeted to mitochondria ^103^. Furthermore, several alternative entry factors to ACE2 and facilitators capable to mediate SARS-CoV-2 infection have been identified ^104– 107^, and they could mediate SARS-CoV-2 caveola-mediated endocytosis. Taken together, it is possible that the diversity of receptors and entry pathways exploited by SARS-CoV-2, together with the fast dynamics of PM-mitochondria communication can obscure the caveolae role that could mediate mitochondria infection.

If caveolae does mediate mitochondria infection by SARS-CoV-2, then relevant questions are the compartments involved and the resulting membrane topology. In this regard, at least two alternatives are possible: a) the first one is that the vRNA alone is translocated through the TOM complex from the cytoplasm or directly from the caveolae after viral and caveola membrane fusion. Indeed, the TOM complex has been already involved in SARS-CoV-2 vRNA presence within mitochondria ^34^, and together with TIM complex are known to mediate RNA import into mitochondria matrix ^85^; b) the second is an odd although feasible possibility. The caveola membrane, fused or not with the viral membrane, could fuse with the MOM, thus delivering into the intermembrane space either the viral particle, or the vRNA in case caveola and viral membranes were pre-fused. In this step TOM20 may play a role ^34^, and could be related with the observation of ACE2 at mitochondria and with TEM showing a close interaction between a SARS-CoV-2 caveola-like vesicles with mitochondria ^54^. Then, the viral particle could fuse its membrane with the IMM and enable vRNA import into mitochondria matrix, although the mechanism seems problematic because S activation to mediate membrane fusion would be required. Alternatively, if only vRNA is translocated into the intermembrane space, then the TIM could help to import it into mitochondria matrix. The replication of SARS-CoV-2 in bacterial cultures seems to support this mechanism ^73^. Mitochondria infection may result in the synthesis of viral proteins having transmembrane domains involved in vRNA replication at mitoribosomes, profiting there the Fe-S clusters synthesized by mitochondria that are required for the RdRp function ^71^. The experiments with protein translation inhibitors in SARS-CoV infected cells suggest that mitoribosomes and eukaryotic ribosomes participate in the synthesis of proteins involved in RNA replication because cycloheximide did not fully blocked it but puromycin did. Further experiments are required to explore these possibilities.

After this review was initially published online in 2022, several publications have further documented the relevance of mitochondria in SARS-CoV2 infection. I apologize with the authors of these works not reviewed here, unfortunately I am unable nowadays to carry out such task for the reasons explained in the acknowledgements section. Nonetheless, in a recent paper, it was observed that cell transfection with nsp3-4 induced DMV-like vesicles that are yield apparently from the ER ^108^. Despite these observations challenge the central idea described in this work and supports the ER origin of DMV, these particular DMV-like vesicles should be studied further, since zER was not apparent and it is presumably an intermediate step for DMV generation according to the ER origin model. Moreover, critical pending observations, described above, still need to be reconciled if the DMV source is the ER.

### Integration into the model of SARS-CoV-2 infection and pending questions

According to the published literature, it seems possible to conceive that SARS-CoV-2 DMV have a mitochondrial origin, through the shedding of MDV as shown in Figure 1. This possibility is supported by different observations reviewed here and would include mitochondria infection by vRNA, that has been observed *in vivo*, *in vitro* and by TEM experiments ^24, 34, 35^. Mitochondria infection occurs through TOM complex function ^34^, but it is not clear whether direct targeting to mitochondria of PM caveolae containing SARS-CoV-2 occur ( Figure 1 question mark 3), as it has been reported with caveolae in myocytes and astrocytes, and has been hinted by TEM in infected cells from patients ^54^. A major advantage of the proposed role of mitochondria in DMV assembly, in comparison with their origin from the ER, is the shortest time to induce DMV, since protein synthesis required to induce ER zippering and bending would not be necessary until later when the ER is infected by vRNA. However, still many questions remain in this scenario, and most probably, previous findings that escaped this review may challenge this hypothesis, that nevertheless pretends to be an integrative proposal to further examine under a new optic DMV origin, function and SARS-CoV-2 replication cycle. Importantly, is it possible that contacts between DMV and other RO-modified membranes could be related to MAMs? Structures that mediate ER-mitochondria interactions and are critical for their function through different mechanisms ^36, 69^.

It is also important to elucidate the steps that may promote MDV shedding after vRNA infection and how these MDV transform into DMV ( Figure 1 question mark 1). The production of ROS, that have been demonstrated after viral infection ^50, 53^, could be the trigger, but this needs to be demonstrated. In addition, it seems possible that other mitochondrial molecules present at MDV/DMV increase viral fitness. Their identification could potentially open new avenues or novel strategies to prevent excessive viral replication and/or severe COVID-19. Interestingly, subpopulations of MDV differ in their proteomic content depending upon the mechanism of induction ^42^.

Another relevant question is the origin of ribosomes that decorate DMV, that could assemble *de novo* with putative mitochondria translocation signals or the action of viral proteins, similar to MOM tethered ribosomes ^84^. These ribosomes are related with the PINK1/Parkin pathway, and it is uncertain whether they are involved in the immediate translation of vRNA after its export from the DMV ( Figure 1 question mark 2).

Also, the identification of a putative FASTKD5 interaction with M protein opens the possibility that within mitochondria, vRNA could be target of maturation, which in turn could optimize viral protein synthesis at mitoribosomes, or when this processed vRNA is exported from DMV. Alternatively, this putative interaction could prevent mitochondria RNA translation. In this regard, codon variation in the human mitochondria genetic code could provide clues that shed light regarding viral protein synthesis by mitoribosomes ^109^.

Another intriguing possibility that should be tested is whether DMS eventually become vesicles with virions inside ( Figure 1 question mark 4). This is because, the faith of DMS is not clear, however, given the topology of their membranes and the nearby synthesis of viral proteins (perhaps in the interior of DMS), it is conceivable that the closed inner membrane becomes the viral membrane, deployed later to the ERGIC.

Also pending is whether CM are the byproduct of DMV or DMS, as it has been proposed ^24, 33^. Instead, CM could be collapsed DMV that exhausted available resources in their vicinity and interior, since CM increase after DMV formation slows down ^33^, and/or debris that remains after DMS assembly. Both mechanisms are consistent with the accumulation of viral proteins at CM. In this regard, it has been shown that coronavirus vRNA synthesized *de novo* colocalize with dsRNA in early stages of infection, but that they segregate in later stages ^110^. Interestingly the same work reported that *de novo* synthesized vRNA is initially concentrated together, but later on it disseminates throughout the cell, possibly as the result of MDV shedding.

 In addition, could this alternative pathway of SARS-CoV-2 cell infection be related with the incomplete or lack of effect of drugs that target the endolysosomal pathway?

All these questions require further research to be answered and would test this complimentary model of SARS-CoV-2 infection of mitochondria and DMV assembly. Nevertheless, it seems clear that a diversity of cellular mechanisms (entry factors and facilitators, endocytosis, cleaving proteases, organelles) are exploited by SARS-CoV-2 to infect cells, replicate and succeed.

Given the pandemic emergency worldwide, a deeper understanding of the cellular mechanisms that are exploited by SARS-CoV-2 to infect cells and replicate seems urgent as it could lead to envisage novel therapeutic targets and alternatives to control or stop the pandemic that today is still enhancing the death toll. The model proposed here for SARS-CoV-2 infection and DMV assembly provides a non-conventional scenario to explore, that could help to treat or prevent SARS-CoV-2 infection and replication, for instance with mitochondria-targeted molecules (i.e chloramphenicol alone or in combination with other drugs; mitochondria-targeted RNA; mitochondria protein/cofactor synthesis and function, etc.), some of which have been identified as candidates to treat COVID-19 ^74^. Indeed, azithromycin, an antibiotic with a similar mechanism of action as chloramphenicol, has been reported to benefit against different viral infections and has also been investigated as a treatment for COVID-19 ^111^. However, mixed results have not allowed to reach a clear conclusion, because different protocols have been employed to assess its effects, many times in combination with hydroxychloroquine or days after infection. However, if the putative mechanism of action, beyond that well-known as antibiotic and those previously reported in immunomodulation, includes the interference of viral protein synthesis through mitoribosomes, then the time window for treatment is critical, since it requires to be administered early during viral replication, that occurs the first days after symptoms onset, as it has been discussed previously ^111^.

## Conclusions

Evidence at morphological, biochemical, molecular and cellular levels reviewed here strongly support that MDV, most probably TOM+, are the precursors of DMV induced by SARS-CoV-2 cell infection. This mitochondrial vesicular intracellular traffic pathway described some years ago is still emerging, reason that perhaps has precluded its consideration in coronavirus infection. Nonetheless, this pathway offers some relevant advantages in comparison with the ER origin mechanism. The main one is that the topological difficulties that implicate the generation of many 3D spherical structures with a double membrane from the 2D rather planar ER are bypassed. In principle, given that MDV are quickly and easily generated through a scission mediated by drp1, this in turn would reduce the time and energy (ATP) required to generate a large number of DMV in a couple of hours. These features are consistent with the early appearance of DMV in infected cells and the subsequent appearance of other membrane modifications of the RO. In addition, the MDV pathway may reconcile the lack or partial overlap of conventional ER markers in DMV, as well as the demonstration of non-conventional ER markers in DMV that have been identified as part of the MAMs proteome. Furthermore, the inconsistent effect of protein translation inhibitors on RNA synthesis in infected cells could also be reconciled if viral protein synthesis in mitoribosomes is considered. Nevertheless, several questions still remain to be investigated to further support or question this hypothesis. Undoubtedly, the confirmation of vRNA within mitochondria, initially anticipated by a modelling approach, demonstrated just few months ago and detected in a previous work through TEM, provide a strong argument in favor of this idea, although a definitive proof is still lacking. On the other hand, the literature reviewed here points towards a substantial role of ER-mitochondria contacts (MAMs) in the genesis of DMV/MDV. Despite it is possible that some findings regarding coronavirus replication cycle that question this proposal may have escaped this review, several lines of evidence reviewed here advocate the origin of DMV from mitochondria.

The current pandemics has resulted in thousands of articles published monthly studying the SARS-CoV-2 or COVID-19. This burst of studies has made clear that mitochondria play a critical, if not central, role in SARS-CoV-2 infection, at the cellular and organismal levels. This discovery has opened new opportunities to tackle SARS-CoV-2 infection and COVID-19 considering the mitochondria biology, that has been studied for decades. Similarly, the mitochondrial origin of DMV/MDV represents an unforeseen chance to develop novel strategies to control viral replication, that in turn should provide better outcomes to COVID-19 patients. More basic and clinical work is required to test these possibilities and putative therapies based in these assumptions.

## Abbreviations

**CM**-convoluted membranes; **DMS**-double membrane spherules; **DMV**-double membrane vesicels; **ER**-endoplasmic reticulum; **ERGIC**-ER-Golgi intermediate compartment; **IMM**-inner mitochondria membrane; **M**-mitochondria; **MAM**-mitochondria associated membrane; **MAVS**-mitochondria antiviral-signaling protein; **MOM**-mitochondria outer membrane; nsp-non-structural protein; **nsp**-non-structural protein; **PM**-plasma membrane; **TIMM**-translocase of the mitochondrial inner membrane; **TOM**-translocase of the mitochondrial outer membrane; **VDAC**-Voltage-dependent anion channel; **VP**-vesicle packet (not shown); **vRNA**-viral RNA; **zER**-zippered ER.

## Data Availability

All data underlying the results are available as part of the article and no additional source data are required.
