# Supplementary material for: Evidence of mitochondria origin of SARS-CoV-2 double-membrane vesicles: a review
Source: F1000Res. 2024 Jul 10;10:1009. Originally published 2021 Oct 5. [Version 3] doi: 10.12688/f1000research.73170.3 (PMC11140301; doi:10.12688/f1000research.73170.3)
Supplement: Peer review history

# Reviewer report: Reviewer response for version 3

**DOI:** 10.5256/f1000research.168837.r363283

**Published:** 2025-02-17

**Authors:** Benjamin Neuman

**Affiliations:** 1 Texas A&M University, College Station, Texas, USA

The introduction of the paper contains several statements that appear to be inaccurate or misleading. For example, NSP3 does indeed form pores, but only in the presence of NSP4. Additionally, the term "N" refers to the nucleoprotein or nucleocapsid, not the nuclear protein. There is substantial evidence supporting the presence of ribosomes on double-membraned vesicles (DMVs), suggesting their origin is not related to mitochondrial membranes. I am unaware of any serious claims within the field proposing such a connection. Moreover, the conflation of DMVs and MDVs, which seems to be a central premise of this review, is not supported by the broader scientific literature. Additionally, the paper expresses surprise that treatments inhibiting host ribosome synthesis, which also harm the host cell, impede virus replication. This is a well-established concept dating back to the 1980s and applies broadly to all viruses. Furthermore, the diagram presented in the paper, which shows double-membraned spherules that are neither open nor connected to a double membrane, appears to be unclear and inconsistent with current understanding.

 The majority of the cited data seems to come from the influx of research conducted during the early stages of the COVID-19 pandemic. While this surge of research was well-intentioned, much of it lacked depth, with some findings proving less impactful upon closer examination. The studies cited in the paper primarily date up until 2020, as expected for a paper first published in 2021. However, in the time since, subsequent studies have failed to corroborate some of the proposed connections. For instance, the hypothesis that DMVs originate from mitochondrial membranes was initially plausible but has not been substantiated by later research.

 Although the paper may offer an imaginative perspective for potential new avenues of research, the connections drawn by the author appear tenuous at best. While creativity is essential in scientific inquiry, this work does not meet the rigorous standards typically expected of scientific publications, as it introduces speculative ideas without sufficient evidence to support them. In scientific practice, caution is critical, as the primary responsibility of researchers is to avoid disseminating incorrect information.

 On a positive note, some of my work was cited in the paper, and for the most part, the references were accurate. This is a small but welcome acknowledgment.

 In conclusion, while this paper is assembled from a range of scientific and quasi-scientific sources, it does not qualify as a comprehensive or rigorous scientific review. It lacks the coherence and critical evaluation expected in such works.

Is the review written in accessible language?

Partly

Are all factual statements correct and adequately supported by citations?

No

Are the conclusions drawn appropriate in the context of the current research literature?

No

Is the topic of the review discussed comprehensively in the context of the current literature?

Partly

Reviewer Expertise:

Coronavirus biology, evolution and discovery with a special focus on DMVs.

I confirm that I have read this submission and believe that I have an appropriate level of expertise to state that I do not consider it to be of an acceptable scientific standard, for reasons outlined above.

# Reviewer report: Reviewer response for version 2

**DOI:** 10.5256/f1000research.140173.r260943

**Published:** 2024-05-30

**Authors:** Md Imam Faizan

**Affiliations:** 1 University of Rochester (Ringgold ID: 6927), Rochester, New york, USA

The manuscript presents an intriguing exploration of the role of mitochondria-derived vesicles (MDVs) in the viral replication cycle, particularly emphasizing the formation of double membrane vesicles. The author has adeptly synthesized a substantial body of research to elucidate this complex process. However, there appears to be an oversight in the literature review, as some pivotal studies that could further enrich the discussion have been omitted. I recommend the inclusion of the following two articles to bridge this gap and enhance the comprehensiveness of the review:

1. Klein S et al, 2020 [Ref 1]
2. Zimmermann L et al, 2023 [Ref 2]

Regarding the graphical elements, the quality of Figure 1 could be significantly improved to better support the narrative. Additionally, the figure legend is currently too extensive and could be streamlined for clarity. Portions of the legend that provide general information could be integrated into the main text to maintain focus on the key findings depicted in the figure.

 While the selection of Transmission Electron Microscopy (TEM) images effectively demonstrates MDV shedding post-viral RNA infection, the visual impact of the paper would benefit from the inclusion of color confocal microscopy images (if any). These images could provide a more vivid and informative representation of the phenomena described, thereby reinforcing the author’s statements with visual evidence.

Is the review written in accessible language?

Yes

Are all factual statements correct and adequately supported by citations?

Partly

Are the conclusions drawn appropriate in the context of the current research literature?

Yes

Is the topic of the review discussed comprehensively in the context of the current literature?

Partly

Reviewer Expertise:

Molecular biology

I confirm that I have read this submission and believe that I have an appropriate level of expertise to confirm that it is of an acceptable scientific standard.

## References

[1] : SARS-CoV-2 structure and replication characterized by in situ cryo-electron tomography. *Nat Commun* .2020;11(1) : 10.1038/s41467-020-19619-7 5885 10.1038/s41467-020-19619-7 33208793 PMC7676268

[2] : SARS-CoV-2 nsp3 and nsp4 are minimal constituents of a pore spanning replication organelle. *Nat Commun* .2023;14(1) : 10.1038/s41467-023-43666-5 7894 10.1038/s41467-023-43666-5 38036567 PMC10689437

# Response

**Published:** 2024-07-04

**Authors:** Pavel Montes de Oca-B

**Affiliations:** IFC-UNAM/INNN-SSA, Mexico

First, I want to deeply thank MD Imam Faizan for reviewing the manuscript. I appreciate very much his judging of this work. Below, I respond to his observations.

 1.- The two references that were suggested are now included in the new version of the manuscript.

 2.- The figure legend was shortened where possible as suggested.

 3.- Given the reasons explained in the acknowledgement section, it is now not possible for me to make changes to the figure. However, it was provided in high resolution and therefore, it must have better detail than the one shown in some versions of the manuscript. Neither, I am able now to review the literature generated in the last two years. I am very sorry for this inconvenience and hopefully in the future I may carry out this task.

 4.- For the same reason, it is not possible now for me to carry out the required experiments to obtain confocal images to test this hypothesis. I am very sorry for this inconvenience and hopefully in the future I may carry out these experiments.

# Reviewer report: Reviewer response for version 2

**DOI:** 10.5256/f1000research.140173.r155519

**Published:** 2022-11-28

**Authors:** Marc Germain

**Affiliations:** 1 Groupe de Recherche en Signalisation Cellulaire and Département de Biologie Médicale, Université Du Québec à Trois-Rivières, Trois-Rivières, QC, Canada

The author addressed my previous comments.

Is the review written in accessible language?

Yes

Are all factual statements correct and adequately supported by citations?

Partly

Are the conclusions drawn appropriate in the context of the current research literature?

Partly

Is the topic of the review discussed comprehensively in the context of the current literature?

Yes

Reviewer Expertise:

Mitochondria, MDV, endoplasmic reticulum, lysosomes

I confirm that I have read this submission and believe that I have an appropriate level of expertise to confirm that it is of an acceptable scientific standard.

# Reviewer report: Reviewer response for version 1

**DOI:** 10.5256/f1000research.76802.r124209

**Published:** 2022-03-16

**Authors:** Marc Germain

**Affiliations:** 1 Groupe de Recherche en Signalisation Cellulaire and Département de Biologie Médicale, Université Du Québec à Trois-Rivières, Trois-Rivières, QC, Canada

In this manuscript, the author argues that the double-membrane vesicles (DMVs) required for coronavirus replication could originate from mitochondria-derived vesicles (MDVs). This is an intriguing possibility that, if true, would open new research avenues. However, there are a number of holes in the argumentation that required to solidify the hypothesis.

1. DVMs are generally assumed to originate from the ER. The author argues that because of the shortcomings of this hypothesis, we should be looking for a different source for these membranes. However, the proposed MDV origin also comes with its own issues and unknown. The strengths and weaknesses of each model should be directly compared to allow the reader to properly assess both.
2. Similarly, the potential role of ER-mitochondria contact sites in viral RNA replication should be better discussed.
3. The description of MDVs does not really reflect the complexity of these vesicles. Several types of MDVs exist, each with its own cargo and destination, and not all of them contain oxidized mitochondrial content destined for degradation in response to mitochondrial stress. What kind of MDVs are envisioned as the source of DMVs (TOM20-positive outer membrane vesicles? Vesicles with oxidized cargo? MAPL-positive MDVs? Other types of MDVs?). Also, MDVs are DRP1-dependent (doi: 10.1038/s41556-021-00798-4).
4. The author makes a compelling point for the involvement of mitochondria in coronavirus infection, but this does not necessarily extend to MDVs for which the evidence is much weaker.
5. It should be made clear in the text that the interactions reported in ref 40 are for the most part putative as they have not been experimentally confirmed. Also, some of the potential interaction need to be more carefully described. For example, TRIM and REEP are large family of proteins with various members having distinct functions and interacting partners.
6. Some references are missing (transfer from PM to mitochondria in minutes, p.7) or wrong (figure 3B is from ref 48, not 46; ref 45 does not discuss viral proteins).
7. How viral RNA or protein synthesis would be targeted to mitochondria is unclear. Ribosomes are recruited to mitochondria because the proteins they are translating contain a mitochondrial-targeting sequence (MTS) that bind to the TOM complex. Are there MTS on the viral proteins targeted to mitochondria?
8. What would be the membrane topology resulting from the caveolin-dependent delivery of viral particles to mitochondria? How would nsp3/4/6 reach the inner membrane? In this context, where would the precursor polyprotein be cleaved to generate the individual proteins?
9. Minor point: in the introduction, it is rather strange to state that different vaccine strategies are under development when, in fact, vaccines have been used for over a year.

Is the review written in accessible language?

Yes

Are all factual statements correct and adequately supported by citations?

Partly

Are the conclusions drawn appropriate in the context of the current research literature?

Partly

Is the topic of the review discussed comprehensively in the context of the current literature?

Yes

Reviewer Expertise:

Mitochondria, MDV, endoplasmic reticulum, lysosomes

I confirm that I have read this submission and believe that I have an appropriate level of expertise to confirm that it is of an acceptable scientific standard, however I have significant reservations, as outlined above.

## References

[1] : MIROs and DRP1 drive mitochondrial-derived vesicle biogenesis and promote quality control. *Nat Cell Biol* .23(12) : 10.1038/s41556-021-00798-4 1271-1286 10.1038/s41556-021-00798-4 34873283

# Response

**Published:** 2022-03-23

**Authors:** Pavel Montes de Oca-B

**Affiliations:** IFC-UNAM/INNN-SSA, Mexico

First, I want to deeply thank Dr. Marc Germain for reviewing the manuscript. Below, each point raised is tackled and appropriate modifications and additions will be included in the following version of the manuscript, that will also include the issues raised by a second reviewer. Beforehand, I apologize if these modifications are not immediately performed in the manuscript, since considering all comments and questions from reviewers as a whole will be much easier, practical, and integrative for a new version of the manuscript.

1. The main concerns with the ER origin of DMV are: the topological needs that such origin implicates and the high energetic cost that such model would require. That is, if one considers the ER origin according to the proposed model described by Mihlec et al., 2021, it seems clear that the movement of ER membranes required to first  form and then close DMV seem to be rather complex, mainly at the closing point, because it is required that a 2 dimensional plane is folded to converge into a single closing point, to then scission the vesicle from the ER, resulting in a sphere (in the case of a single DMV). In this regard, it seems more plausible the mitochondrial origin, because it only requires an evagination and a scission from an organelle that already has a double membrane (that in terms of topology, pose a huge problem with the ER origin). The scission of vesicles continuously occurs in mitochondria under basal conditions and is increased under stressful conditions. Interestingly, such stress may be elicited by mitochondria infection with vRNA, that has been recently demonstrated (Shang et al., 2022). Theis mechanism is less contentious than a complicate series of movements and foldings from a 2-dimensional plane, that most probably would require the involvement of the cytoskeleton and molecular motors. On the other hand, the involvement of molecular motors, required to move membranes, implicates the use of energy, and considering the complicate foldings and movements required in the ER model, as well as the large number of DMV that are induced after infection, then the required energy would be also considerable and could be a limiting step (Interestingly, the SARS CoV-2 infection induces glycolysis and importantly reduces OXPHOS). This complexity contrasts with the rather simple and common evagination and scission from the mitochondria, mechanism of vesicle assembly that occurs all the time, not only at mitochondria level, but also at the plasma membrane and other intracellular organelles, the classical mechanism of vesicle formation. In this regard, the ER origin of DMV would implicate that a completely different mechanism of vesicle formation, not observed in cells to my knowledge, is induced by the virus. Despite this is not impossible, from the evolutionary point of view, it seems improbable that a unique, complex and costly mechanism has been selected along with the efficient infective coronavirus, over the common cellular mechanism of vesicle formation.
  Finally, it is intriguing that the ER origin of DMV is advocated almost unquestionably when it has been reported the absence of conventional ER markers in DMV, such as protein disulfide isomerase (PDI) or calreticulin (Snijder, et al. 2006; Ulasli et al, 2010; Oostra, et al 2007). In contrast other non-conventional markers such as calnexin or RTN3 have been observed (Hackstadt et al., 2021; Cortese et al. 2021). Nevertheless, and interestingly, these molecules are part of the proteome of mitochondria-ER contacts (Cho et al., 2017).
  These considerations will be included in the reviewed version of the manuscript.
2. Possibly, these contacts are involved in favoring immediate protein synthesis after vRNA exit from the DMV. Nevertheless, they could also provide Ca2+ to the DMV for RNA synthesis, or lipids to enable DMV growth. Also, it is known that mitochondria-ER contact disruption impairs organelle recycling, that FIS-1 recruits DRP for mitochondria fission, and that they are involved in apoptosis, mitophagy and autophagy induction (Lee and Min, 2018). The regulation of these functions could also be relevant for SARS CoV-2 replication cycle. This requires further research to be solved. These considerations will be included in the reviewed version of the manuscript.
3. Necessarily, in my opinion, it would be double membrane vesicles, a key feature of DMV, and it is feasible that they are those containing the TOM machinery to translocate specific proteins into de DMV/MDV. Whether they may content oxidized cargo needs to be evaluated, because the infection reduces OXPHOS then, perhaps not so many oxidized molecules are generated. The mechanism of induction could be other than oxidation within mitochondria, possibly vRNA infection of mitochondria itself, that has been recently demonstrated for SARS CoV-2 (Shang et al., 2022). The dynamics of OXPHOS decrease and glycolysis induction may shed some light to answer this question. The presence of MAPL/MULAN could occur because it is an interactor of the viral proteome, that nevertheless seems to be non-abundant in MDV.
  Regarding the role of DRP it seems indeed contra intuitive, since DRP has been reported to be degraded through the action of the viral protein ORF9b (Shi et al., 2014). In this regard it is possible that ORF9b is synthesized later after DMV generation, that occurs early after infection. That is, mitochondria infection induces DMV early, and later after protein synthesis, from de novo synthesized vRNA in DMV, ORF9b induces DRP degradation. Importantly, since the experiments that demonstrated DRP degradation by ORF9b were performed in a cell line that constitutively expresses ORF9b, it is conceivable that this model differs from ORF9b expression in viral infected cells. Furthermore, despite this was an ORF9b overexpression system, DRP was not fully degraded, 30% of total DRP according to authors was not degraded. Thus, the dynamics of DRP degradation induced by ORF9b requires to be confirmed in real-viral infected cells, because even proteasome degradation could be impaired given the high expression of viral proteins.
  These considerations will be included in the reviewed version of the manuscript.
4. I do agree, since the idea that MDV could originate DMV has not been demonstrated and this manuscript only collects the evidence supporting this idea, more research is required to test the possible involvement of the MDV pathway. Also, it is required to test whether MDV differ from DMV at the molecular level and in their genesis, if it is the case that they are derived from mitochondria. The fact that it has recently been demonstrated that mitochondria is infected by SARS CoV-2 vRNA advocates for MDV as DMV precursors (Shang et al., 2022).
5. Ok, this will be stated more clearly in the following version of the manuscript. Also, the specific interaction of TRIM and REEP with viral molecules will be more detailed and analyzed.
6. Yes, sorry for the mistake. This has been corrected and all references checked.
7. According to this idea, vRNA could be translated full or in part (at least those proteins required for RdRp assembly) at mitochondrial ribosomes within the mitochondria matrix, that are closer to prokaryotic ribosomes. Nevertheless, surface MOM ribosomes could translate de novo vRNA emerging from the DMV. As far as I am aware, no MTS has been reported in viral proteins, although I am not sure that they have been looked for. Nevertheless, for proteins that are translated within the mitochondria no MTS would be necessary.
  These considerations will be clarified in the reviewed version of the manuscript.
8. The delivery of complete viral particles into mitochondria seems improbable, as it would require the escape of the full viral particle from the vesicle, as well as from the fusion mediated by Spike that enables vRNA translocation to the vesicle external compartment. One odd topological possibility exists nevertheless, the fusion of caveola to the MOM, that would enable the virus fuse with the IMM. However, this is only a topological possibility that seems difficult given the protease and pH needs required to activate Spike protein fusion machinery that may not be fulfilled at the intermembrane space. Rather, I would favor the idea that the vRNA could be translocated into mitochondria, either directly from the caveolae, or after an initial translocation to the cytoplasm. Importantly, vRNA has been demonstrated this year within mitochondria matrix (Shang et al., 2022). These scenarios require further investigation, since plasma membrane-mitochondria interactions have been somewhat disregarded (Montes de Oca B, 2021).
9. You are right, I wrote the initial draft before April 2021, they are still in clinical trials III and IV. This will be corrected in the manuscript.

# Response

**Published:** 2022-11-21

**Authors:** Marc Germain

**Affiliations:** Université du Québec à Trois-Rivières, Canada

Thank you for the clarifications. Do you think that the synthesis of viral proteins on mitochondrial ribosomes would be affected by the fact that the mitochondrial genetic code is slightly different from the standard code?
